# Digitalization to improve tax compliance: Evidence from VAT e-Invoicing in Peru^☆^

**DOI:** 10.1016/j.jpubeco.2022.104661

**Published:** 2022-06

**Authors:** Matthieu Bellon, Era Dabla-Norris, Salma Khalid, Frederico Lima

**Affiliations:** The International Monetary Fund, United States

**Keywords:** Electronic invoicing, VAT, Tax compliance, Digitalization

## Abstract

This paper examines the impact of switching from paper to electronic invoicing on firm tax compliance and performance using quasi-experimental variation in the roll-out of VAT e-invoicing in Peru. We find that e-invoicing increases reported firm sales, purchases and VAT liabilities by over 5 percent in the first year after adoption. The impact is concentrated among small firms and sectors with higher rates of noncompliance, suggesting that e-invoicing enhances compliance by lowering compliance costs and strengthening deterrence. However, we also find that existing stocks of VAT credits were used to offset the reform’s positive effects on VAT collection, suggesting that digital tools such as e-invoicing would need to be complemented by other reforms to improve revenue mobilization.

## Highlights

• Following electronic invoicing reform in Peru, firms report higher VAT obligations.
• Small firms and sectors with low tax compliance show strongest effects of policy.
• Suggests effects of e-invoicing operate through improved monitoring and deterrence.
• Some firms exit when e-invoicing requirements are announced; reveals adoption costs.
• Complementary reform needed to unlock VAT collection increase to full potential.

## 1 Introduction

Digitalization is transforming how tax administrations operate by vastly increasing their ability to collect, process and monitor tax information. A striking example is electronic invoicing (e-invoicing), which allows for the automated transfer of billing information between firms and the tax authority. Drawn by its potential to improve tax compliance and reduce costs, more than 50 countries around the world have already adopted e-invoicing, including ten countries in Latin America and the Caribbean region (Barreix and Zambrano, 2018).

By replacing more cumbersome paper-based processes, e-invoicing promises multiple benefits for firms and the tax authorities alike, including lower administrative and compliance costs, better integration of billing and payment systems, improved accuracy and information security, and easier access to short-term financing. For tax administrations, e-invoicing also delivers real-time information that could be used to strengthen and automate compliance checks. However, despite its widespread adoption, there is still limited empirical evidence on how e-invoicing affects firm compliance and performance. This paper addresses this gap by using administrative tax data and quasi-experimental variation in the mandatory roll out of value-Added Tax (VAT) e-invoicing in Peru.

The electronic transmission of invoice information in Peru required a substantial overhaul of tax administration and taxpayer IT capabilities. As a result, e-invoicing was introduced gradually, with the first reform waves focusing on larger firms and priority sectors, while smaller firms were given more time to adopt the new electronic system. Our identification strategy exploits this sequential introduction of the reform to estimate the causal impact of VAT e-invoicing on firm performance and compliance.

We use confidential administrative data provided by the Peruvian tax authority (SUNAT) to conduct our analysis. Our quarterly panel dataset covers all small, medium and large firms registered to pay VAT in Peru between 2010 and 2017, and includes detailed information on firm sales, purchases, employment, and taxes. To avoid composition biases, our analysis focuses on a balanced sample panel consisting of over 53,000 firms that were mandated to adopt e- invoicing between 2014 and 2018, and which account for over 85 percent of domestic VAT collections in Peru.

We conduct the analysis in four steps. First, we estimate the impact of the e-invoicing reform on sales, purchases and VAT liabilities across firms. We find that being mandated to adopt e-invoicing (an “intent-to-treat” effect) increases reported taxable sales and purchases by 7.4 and 5.5 percent in the first year, respectively. This impact grows over time, starting from the mandatory date of adoption. The resulting increase in taxable value added does not seem to be associated with a commensurate increase in labor inputs, suggesting that it is driven by an increase in the share of output that is reported to the tax authorities. Furthermore, the increase in sales and purchases does not translate into a one-for-one increase in VAT collections. Instead, we find that existing stocks of VAT credits allow some firms to offset VAT liabilities and, consequently, to lower VAT payments in the first year of e-invoicing.^1^

Second, we examine how these estimated impacts vary across firms. We show that the positive impact of e-invoicing on reported sales and tax is driven primarily by smaller firms. Specifically, we find that VAT liabilities increase by 11.6 percent in the first year after adoption among SMEs, while the effect among large firms is smaller and not statistically significant. Back of the envelope calculations suggest that the reform resulted in a net increase of 6.7 percent in total reported VAT obligations among SMEs and large firms but a net decline of 3.1 percent, driven by payment decline in SMEs and large firms that hold stocks of VAT credits.

In addition, we find that the reform had a larger impact in sectors that traditionally suffer from low compliance, such as construction and business services. Firms in these sectors respond more strongly to e-invoicing adoption, suggesting that e-invoicing affects firm behavior in part by fostering greater compliance, possibly because of the perceived threat of greater scrutiny. We also find that firms in these sectors are more likely to exit once the e-invoicing reform was announced.

Third, we show that there is no evidence of anticipation effects before the e-invoicing adoption deadline for taxable sales and purchases, and VAT variables. We argue that this is consistent with incentives that do not change before e-invoicing is effectively adopted. However, we do find evidence of increased rates of firm exit following the announcement of e-invoicing requirements but before adoption deadlines. This pattern is consistent with some firms preferring to exit rather than improve compliance or risk being detected. Alternatively, this pattern would also be consistent with technology adoption costs and expected costs of non-adoption that are too large for some firms.

Finally, we find that the rate of e-invoicing adoption increases steadily around the mandatory dates of adoption in every mandated group. This suggests that being mandated into e-invoicing is a strong instrumental variable for studying the average treatment effect of e-invoicing adoption. The result of instrumental variable (IV) regressions are qualitatively similar and quantitatively larger than the intent-to-treat effects. We find that actual e-invoicing adoption by firms increased their reported sales, purchases, and VAT liabilities by over 12 percent.

To account for the possibility that omitted variable bias affects our difference-in-differences strategy, we employ firm fixed effects to control for time-invariant firm characteristics and quarter fixed effects to control for common shocks across all firms. We also include firm-specific trends to control for time-invariant differences in growth rates across firms. Our dynamic specification allows us to evaluate the precise timing of the treatment effects relative to the announcement dates of e-invoicing requirements and to the adoption deadlines, and establish quarterly parallel pre-trends, to ensure that results are not driven by other coincident changes in the economy.

This paper contributes to several strands of the literature on tax compliance. First, our research contributes to the ongoing research on policy responses to tax evasion (see Slemrod (2019) for a review). In particular, our work reinforces results from studies investigating the importance of third-party information reporting on tax compliance, whereby greater information on taxpayer transactions yield fewer avenues for tax noncompliance (Mittal and Mahajan, 2017, Slemrod et al., 2017, Naritomi, 2019, Pomeranz and Vila-Belda, 2019). Our work is also related to the impact of audit probability on taxpayer behavior (Slemrod et al., 2001, Alm et al., 2018), with the deterrent effect of e-invoicing deriving from higher threat of audit as a result of improved ability to identify noncompliance.

In addition, our research is linked to the broader study of how digital technologies can enhance governance and public sector efficiency (Gupta et al., 2017). For instance, e-procurement has been shown to improve infrastructure provision in India and Indonesia through improvements in quality and reduction in delays respectively (Lewis-Faupel et al., 2016). Similarly, Banerjee et al. (2017) find sizeable reductions in leakages from the world’s largest workfare program (NREGS in India) with the introduction of electronic funds flow management.

Finally, we contribute to the growing literature that examines the impact of digital technologies on tax administration, including not only e-invoicing (Ramirez et al., 2018, Bergolo et al., 2018, Artana and Templado, 2018, Castro et al., 2016, Lee, 2016), but also the electronic submission of tax returns or e-filing (Yilmaz and Coolidge, 2013, Kochanova et al., 2016, Okunogbe and Pouliquen, 2018) and the use of electronic sales registry machines (Eissa and Zeitlin, 2014, Ali et al., 2015). In this respect, our work is closest to Fan et al. (2020) who find large declines in VAT deductions and significant increases in VAT payments following a reform in China which introduced digitally encrypted invoices for VAT filing. There are several important differences in our approach and findings. First, Fan et al. (2020) focus only on manufacturing firms, while we use administrative data on all VAT paying firms. Additionally, our identification strategy leverages the sequential rollout of the e-invoicing reform in Peru whereas Fan et al. (2020) exploit variation in exposure intensity across firms given the simultaneous implementation of the reform in China. In contrast with the large impacts found by Fan et al. (2020), we find a much smaller aggregate impact of e-invoicing, with effects concentrated among smaller firms and partly offset by existing stocks of VAT credits. This suggests that the impact of e-invoicing may vary with noncompliance patterns across countries or with the magnitude of deterrent effects associated with specific reforms.

The remainder of the paper is organized as follows. Section 2 describes the reform timeline, while Section 3 presents the dataset and stylized facts. Section 4 outlines the empirical approach, and Section 5 discusses the main results. The last section concludes.

## 2 The e-Invoicing Reform in Peru

### 2.1 Electronic and paper invoicing

E-invoicing is the transfer of invoice information between firms and their suppliers through digital means. Unlike traditional, paper-based invoices, e-invoices contain billing and payment data in a machine-readable format that can be imported directly into account payable systems and shared automatically with third parties, including the tax authority. When adopted broadly, e-invoicing can have several potential advantages, including improvements in VAT compliance, formality and firm productivity. However, despite being available to firms in Peru since the mid-2000s, voluntary adoption had remained low. This led the Peruvian tax authority to announce a multi-stage plan in 2013 to permanently switch from paper to e-invoicing, which is the reform we study in this paper.

For firms, the transition to e-invoices offered several potential benefits. First, e-invoices could be handled and processed more efficiently than paper invoices. In Peru, as in other countries, paper invoices were associated with significant costs, including printing, postage, delivery and archiving physical copies. E-invoices brought sizable savings, with an estimated cost reduction from $0.56 per paper invoice to $0.20 per e-invoice for SMEs, and even lower costs for micro firms. It also allowed a better integration of invoicing with accounting, procurement, and payment systems, reducing mistakes from data entry and processing paper invoices. And by storing invoice information in a more secure and accessible way, factoring receivables using e-invoices became much more appealing, making it easier for Peruvian firms to obtain short-term financing.

The tax authority also expected that e-invoicing would improve compliance. There was a strong belief that e-invoicing would reduce opportunities for VAT fraud, including from under-reported sales (e.g., not reporting transactions or presenting the same invoice to more than one buyer) or overstated deductions (e.g., issuing fake invoices to simulate purchases or reporting purchases unrelated to business operations). These types of fraud were prevalent with paper invoicing, since it was impossible for the tax authority to cross-check the more than 300 million invoices issued every year in Peru. Equally as important, e-invoicing was seen as a way to streamline the tax payment process and reduce compliance costs.^2^ Anecdotal evidence from discussions with private-sector organizations suggests that firms also believed that e-invoicing would reduce the likelihood of receiving substantial fines when audited. Apart from tax compliance, e-invoicing data was also seen as a means to improve the tax authority’s understanding of cross-sectoral linkages and ability to analyze economic activity in real-time and project revenue trends.

To facilitate the transition to e-invoicing, the tax authority gave firms several options on how to issue e-invoices. Larger issuers could develop their own e-invoicing systems (meeting certain technical and regulatory specifications), contract with authorized third-party systems, or use a free software application developed by the tax authority. Firms that issued fewer invoices, like most micro firms, would typically rely on a free online portal set up by the tax authority (SUNAT - Operaciones en Línea - SOL). In all cases, the e-invoice information was shared automatically with the tax authority. In addition, once they transitioned to e-invoices, all firms were expected to remain e-issuers permanently, with paper-based invoices allowed only in exceptional circumstances (e.g., if there was an internet outage).^3^

### 2.2 Timing of the reform

Since it was recognized early on that switching to e-invoicing would create significant adjustment costs for taxpayers and the tax administration, including updating IT capacity and staff training, the e-invoicing transition was introduced in a gradual and staggered manner. Firms were assigned into reform waves with different deadlines for e-invoicing adoption, with selection criteria based on administrative classifications related to size and compliance factors. Larger firms were required to adopt e-invoicing earlier, as they represented a larger share of VAT revenue and had more capacity to update their IT systems.^4^ The tax administration also prioritized e-invoicing adoption by taxpayers with a record of poor tax compliance since e-invoicing was believed to have a stronger deterrent effect and would facilitate the monitoring of their transactions.

**Fig. 1:**
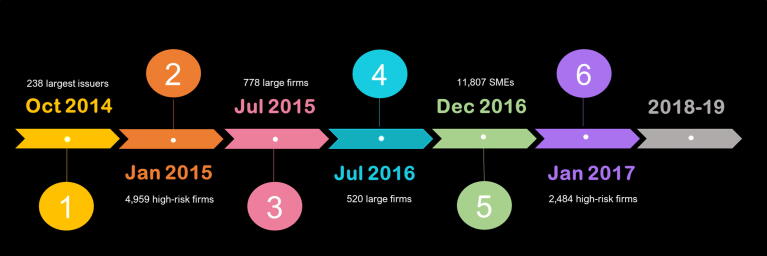
Timeline of e-Invoicing Adoption Waves in Peru. **Note**: This figure illustrates the stages of e-invoicing adoption in Peru. Reform waves are identified by their original adoption deadline.

The first reform wave began in 2014 and included 238 firms among the largest issuers of invoices in Peru, such as large manufacturing, mining and financial firms. The original deadline for this wave was October 2014, but it was later extended to April 2015 and then to August 2015 to give taxpayers additional time to comply.^5^ The second wave comprised 4,959 firms that had been caught in fraudulent transactions (Operaciones No Reales - ONR) during tax audits, and, therefore, were considered as high risk of tax evasion.^6^ These firms were required to switch to e-invoicing starting from January 2015. (see Fig. 1).

**Fig. A.1:**
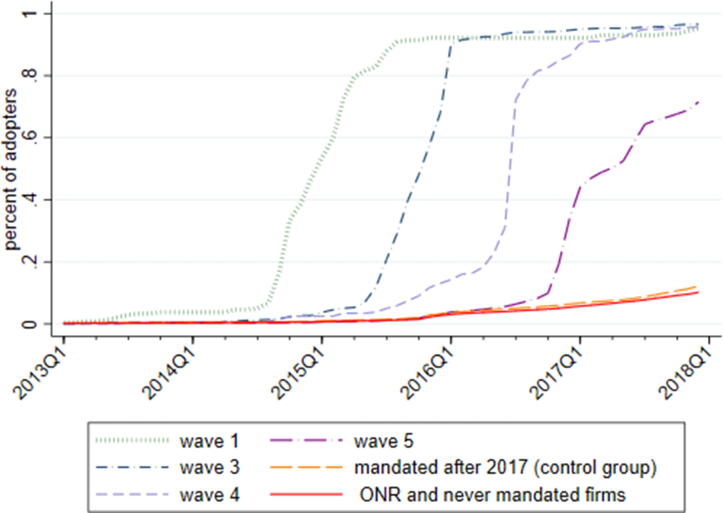
e-Invoicing Adoption Rates by Wave. **Note:** This figure shows the rate of e-invoicing adoption by wave using data from SUNAT. Firm waves are defined based on adoption deadlines imposed by SUNAT. The control group consists of firms that were assigned adoption deadlines after 2017. The last group consist of wave 2 and wave 6 “ONR” firms that were caught in fictitious or fraudulent transactions before their assignment to a wave and of firms that were not yet assigned a specific deadline by 2017.

**Fig. A.2:**
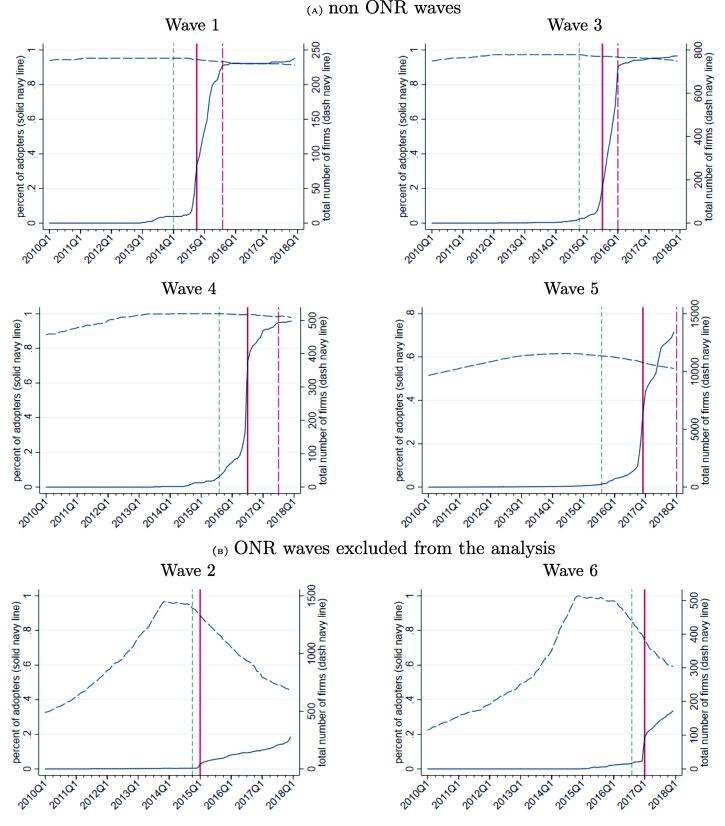
Adoption Rates around First Announcement Dates, Initial and Final Deadlines. **Note:** The number of firms (dash curve) and the fraction of them (solid curve) that adopts e-invoicing are plotted over time. For each wave, dash vertical grey lines indicate the first announcement date. The solid red lines indicate the deadline announced initially. The dash red lines indicate the final revised adoption deadlines. ONR firms are firms that were caught in fictitious or fraudulent transactions.

Reform waves in the next two years continued to focus on larger firms. A group of 778 large firms was required to adopt e-invoicing starting from July 2015 (later extended to January 2016), while a further 520 large taxpayers was given until July 2016 (later extended to July 2017) to make the switch. This staggered selection of firms reflected different vintages of the large firm database maintained by the tax administration, and in both cases the original deadlines for e-invoice adoption were also extended (see Fig. A.1, Fig. A.2 in appendix).^7^

As shown in Table A.1, the first waves of e-invoice adoption included the largest contributors to sales, taxable value added and VAT collections. On average, firms in the October 2014 reform wave reported annual sales of $400 million and employed 1,700 workers each, while those in the July 2015 and July 2016 waves had sales of $50 million and $30 million, and employed 680 and 275 employees each, respectively. These firms were also more likely to be exporters and subject to special VAT withholding regimes.^8^ Together, the firms in the first four waves represented just over 54 percent of total taxable value added and 27 percent of employment in our database.

The next wave (wave 5) focused on expanding e-invoicing to small and medium size firms. A group of 11,807 firms was drawn from the tax administration’s registry of significant taxpayers at the regional and provincial levels and given until December 2016 to switch to e-invoicing. However, this deadline was subsequently extended to July 2017, and then to January 2018.^9^ On average, these firms were smaller in size compared to firms in previous waves, with average annual sales of $5 million and about 100 workers each. However, it still included several larger firms with similar characteristics to firms in earlier waves. Thus, as a group, wave 5 firms account for a large share of economic activity, representing over 20 percent of value added and 30 percent of employment in the database.

A second group of 2,484 firms that had been caught in fraudulent transactions was also required to adopt electronic invoicing starting from January 2017, mainly consisting of firms that had shown poor tax compliance in subsequent audits. In the analysis of e-invoicing that follows, we exclude these firms (waves 2 and 6) for two reasons. First, it is difficult to separate the impact of e-invoicing from the impact of the tax audits and increased monitoring these firms were subject to. Second, the observed e-invoicing adoption rates among these waves did not exceed 20 percent, as opposed to rates over 80 percent for the other waves, reflecting very high exit rates after the e-invoicing requirement was announced.^10^

Starting in 2018, reforms focused on extending e-invoicing to a larger number of small firms, the majority with annual sales between $0.2 and $5 million. Starting from January 2018, the e-invoicing requirement was extended to 4,741 high-risk firms, 4,550 agents of the Retention and Perception withholding regimes 11, and 943 larger firms.^12^ In May, e-invoicing became mandatory for 11,573 small firms that were registered as government suppliers or included in the audited register of inspected goods. In August, the e-invoicing requirement began to apply to 13,837 firms in the manufacturing, construction, hotel and restaurant sectors, and from November onwards to all remaining 54,703 firms with annual sales over $0.2 million. The implementation of e-invoicing across remaining firms was planned from 2019 onwards.

**Fig. 2:**
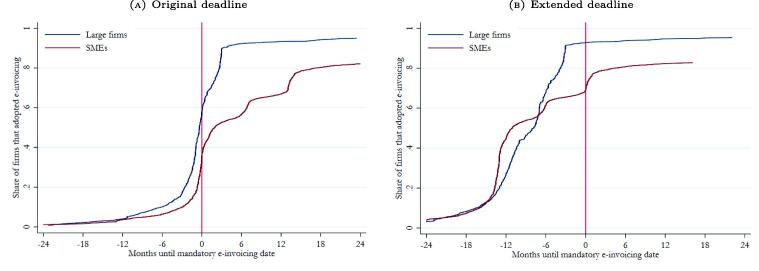
e-Invoicing Adoption Rates Across Waves. **Note:** This figure shows the e-invoice adoption rates across waves, using data from SUNAT. The left panel shows adoption rates relative to the original deadline, while the right panel shows adoption rates relative to the extended deadline (final deadline). The month in which e-invoicing would become mandatory is defined as time 0. The blue lines represent all the firms in wave 1, 3 and 4, while the red lines represent all wave 5 firms.

Fig. 2 shows the rate of e-invoice adoption as firms reached the deadlines set by the tax administration. While adoption rates increase gradually and then spike just before the deadline was reached, they remained between 40 and 60 percent, suggesting that many taxpayers were unable or unwilling to comply with the e-invoicing requirement on time. Deadlines were then extended, by about one year on average, to give firms additional time to comply.^13^ The right panel of Fig. 2 shows that this was a good strategy, since there was typically a high level of compliance by the time the final deadlines were reached. In fact, we see a gradual build up in adoption rates before the final deadline.^14^

While adoption rates are high after the e-invoicing deadline is reached, they never reach 100 percent for any of the reform waves. This reflects the difficulties faced by firms to complete the transition to e-invoicing, even among larger firms. In the analysis that follows, we therefore distinguish between the effects of the reform (mandating firms to adopt e-invoicing whether these firms complied or not) and the actual adoption of e-invoicing by mandated firms.

Finally, we note that during the period we analyze (2013–2017), the tax authority did not adopt any significant changes in its compliance risk management strategy. Consequently, any effects of the reform observed during the period of the study are not confounded by changes in actual monitoring or audit activities of the tax authority. Instead, they would derive purely from the e-invoicing reform and the resulting increase in the probability of tax evasion detection.

## 3 Data and stylized facts

**Fig. 3:**
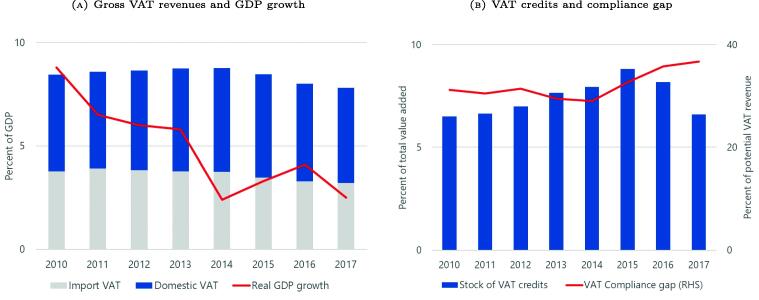
VAT Trends in Peru. **Note:** The left panel plots gross VAT revenue in percent of GDP, distinguishing between domestic and import VAT. The growth rate of real GDP is shown in red. The right panel shows the total stock of VAT credits at the end of each year, expressed as share of total firm value added, and the red line shows the VAT compliance gap (Keen, 2013).

The VAT is the major source of revenue in Peru, accounting for over half of the country’s gross tax revenue. The standard VAT rate was 18 percent during the period we analyze. As shown in Fig. 3, the period during which e-invoicing was introduced was marked by a decrease in VAT revenue from 8.8 percent of GDP in 2014 to 7.8 percent of GDP in 2017. This decline coincided with a slowdown in economic activity after 2014, an increase in the VAT compliance gap, and a marked decrease in the stock of outstanding VAT credits.^15^ These credits are the result of VAT paid on inputs that was not refunded in the year they were incurred, and that are then carried over to subsequent years, when firms can use them to offset future tax liabilities.

We use monthly administrative tax data covering all small, medium and large formal Peruvian firms registered to pay VAT over the period 2010 to 2017. The dataset does not cover two important groups of firms. First, it does not cover micro firms with less than 150 UIT (about $175,000) in annual sales, as these firms were not targeted by the e-invoicing reform during the period we analyze.^16^^,^^17^ Second, it does not cover informal firms since these firms are not registered with the tax authority. Because of these coverage restrictions, the impacts of e-invoicing that we estimate are not directly applicable to these types of firms. Nonetheless, because most micro and informal firms in Peru are very small and make only a small contribution to aggregate value added, our dataset of nearly 200,000 firms retains a broad coverage, accounting for 53 percent of GDP and 95 percent of VAT collections in 2013.^18^

We exclude from our analysis firms that were caught in fraudulent transactions (waves 2 and 6) and the very small firms that were required to adopt e-invoicing only after 2018. Our sample selection strategy ensures that we are comparing firms that are reasonably similar in terms of size and administrative characteristics by evaluating firms that are mandated against those that are yet to be mandated.

We collapse the dataset from monthly to quarterly frequency to facilitate the analysis. In addition, to focus on changes within firms and to avoid composition bias, we also create a balanced panel sample that excludes firms that enter or exit during the sample period (i.e., firms with no reported sales in a given quarter). This balanced dataset includes approximately 53,000 firms that account for 85 percent of VAT collection. As shown in Table A.1, Table A.2, Table A.3, Table A.4 in appendix, the balanced sample remains representative of the original dataset, since average firm characteristics and the distribution of firms across sectors and risk categories by wave are similar across both datasets.

## 4 Empirical approach

We exploit the staggered adoption of e-invoicing to assess its impact on firm performance and VAT compliance. This approach compares the change in outcomes for firms that have been mandated to adopt e-invoicing (the treated group) relative to firms that have not yet been mandated (the control group). Since there were no significant changes in SUNAT’s compliance risk management strategy during the years we study, this approach isolates the impact of the e-invoicing reform separately from any changes due to increased monitoring and audit effort. We specify our panel difference-in-differences model as a fixed effects linear regression:

(1) $$Y_{i,t}=\alpha_{i}+\delta_{t}+\eta_{i}t+\beta \times I\left({\mathrm{Treat}}_{i,t}=1\right)+\gamma D_{i,t}+\varepsilon_{i,t}$$

The dependent variable is a firm-level outcome such as taxable sales, taxable purchases or tax payments, and the coefficient β captures the treatment effect of being mandated to adopt e-invoicing. The indicator variable $I\left({\mathrm{Treat}}_{i,t}=1\right)$ takes on a value of one in the quarter that a firm is mandated to adopt e-invoicing, and the first four quarter after that. We focus on the first year after treatment since we observe all treated groups for at least the first four quarters. Our specification also includes an indicator $D_{\mathrm{it}}$ for the fifth and following quarters after the date of mandatory e-invoicing adoption to independently capture treatment effects more than one year following date of treatment. However, since this indicator only captures treatment effects for a select subset of treated firms for which a longer period of treatment is available in the dataset, we do not use this variable as the focus of our analysis.

The model also includes time fixed effect $\delta_{t}$ to control for shocks common to all firms, such as changes in commodity prices or monetary policy, a firm fixed effect $\alpha_{i}$ to control for time invariant firm characteristics, a firm-specific linear time trend $\eta_{i}t$ to control for heterogeneity in growth paths across firms. These firm specific trends allow us to control for time-invariant differences in growth rates across firms and would correspond to firm fixed effects in a specification in growth rates. Without these trends, the estimation would incorrectly impute post-treatment changes resulting from time-invariant differences in growth rates between the treated and control group to e-invoicing requirements. Standard errors are clustered at the firm level.

Even though the original deadlines for mandated adoption were later extended, we use the original deadlines for our identification strategy. From the firms’ perspective, the original deadline was the relevant constraint, as reflected by the fact that a large proportion of firms adopted at the time of the original deadline. Moreover, given that the transition to e-invoicing requires substantial administrative and procedural changes within the firm, even those firms that had not begun to issue e-invoices at the time of the original deadline would have made progress towards operationalizing e-invoicing, particularly if they are unable to anticipate the provision of an extension. Focusing on the original deadlines allows to capture all these changes.

The identifying assumption in this specification requires parallel trends between the control and treated groups prior to treatment, such that the β coefficient represents the impact of treatment as opposed to differential pre-trends. To test this assumption, we also estimate a dynamic panel difference-in-differences specification which allows us to conduct a pre-trend analysis for treated and control groups and explore the evolution of the treatment effect over the quarters following the mandated date of adoption:

(2) $$Y_{i,t}=\alpha_{i}+\delta_{t}+\eta_{i}t+\underset{t}{\sum }\beta_{t}\times I\left({\mathrm{Treat}}_{i,t}=1\right)+\gamma D_{i,t}+\varepsilon_{i,t}$$

In this specification, the $\beta_{t}$ coefficients capture the dynamic impact of treatment in 6 quarters before and 4 quarters after the mandated date of e-invoicing adoption for a firm, setting the reference period as the quarter before the reform was mandated (i.e. t=-1). Parallel pre-trends require that the $\beta_{t}$ in the pre-treatment period be statistically insignificant, implying no observed differences between the control and treated groups prior to the treatment date. However, given that the date of mandated adoption is pre-announced, some anticipation effects in the quarter leading up to mandated adoption cannot be ruled out.

Since there is imperfect compliance to the e-invoicing reform among mandated firms, the $\beta_{t}$ estimates represent the “intent to treat” (ITT) effect. Moreover, firms that are not mandated into e-invoicing may still adopt e-invoicing voluntarily. While the ITT is considered the policy-relevant parameter given that policy makers cannot force or prevent adoption, we also estimate the Local Average Treatment Effect (LATE) which represents the impact of being mandated into e-invoicing by compliers only.

To estimate the LATE, we use an indicator for being mandated into e-invoicing as an instrumental variable for predicting actual compliance to treatment. Specifically, we estimate a two-stage least squares model where the first stage uses treatment assignment to predict compliance and the second stage uses fitted estimates from the first stage to predict treatment effects:

(3) $$A_{i,t}={\overset{\sim }{\alpha }}_{i}+{\overset{\sim }{\delta }}_{t}+{\overset{\sim }{\eta }}_{i}t+\theta \times I\left({\mathrm{Treat}}_{i,t}=1\right)+\overset{\sim }{\gamma }D_{i,t}+u_{i,t}$$

(4) Yi,t=αi+δt+ηit+βA^i,t+γDi,t+εi,t

where $A_{i,t}$ and A^i,t represent respectively an e-invoicing adoption indicator and the probability of adoption estimated in the first stage. The treatment indicator is only activated for the first year following the mandated quarter of adoption, as in the baseline specification. One note of caution with respect to our LATE estimates is that we do not differentiate between noncompliers and late compliers and assume no change in behavior along other dimensions (reporting of sales, purchases) among this latter group at the time of treatment. However, firms could change their behavior at the time of their mandated date of adoption, even in the absence of having adopted the e-invoicing mechanisms. This could be in anticipation of the eventual switch or owing to the higher threat of audit for being noncompliant. This could bias the LATE downwards assuming the behavior of noncompliers after the treatment date is like the behavior of compliers. The ITT estimate does not suffer from this bias since all firms are considered treated following the mandated date of adoption, regardless of actual adoption, although it might suffer from another bias by not accounting for compliance among untreated firms. However, regardless of the estimator considered, our model is biased against finding a treatment effect and therefore is a conservative lower bound on the potential treatment effect.

## 5 Results

### 5.1 Baseline

We start by estimating the difference-in-differences specification in Eq. 1 using the balanced panel sample. All dependent monetary variables are expressed in constant 2014 Peruvian soles and transformed using the inverse hyperbolic sine function.^19^ Thus, coefficient estimates can be interpreted as percentage deviations from pre-treatment levels.^20^ The main regressor is a treatment indicator that is equal to one in the quarter that e-invoicing became mandatory and in the following four quarters, so that the estimated coefficients represent the average percentage change in the first year of e-invoicing.

The results are presented in Table 1. The first two columns show that taxable sales and purchases are significantly higher among treated firms after the e-invoicing reform, with an average increase of 7.4 and 5.5 percent, respectively. While an increase in taxable sales is expected, the increase in taxable purchases is more surprising, since one motivation of the e-invoicing reform was to reduce fake invoices. The positive impact on taxable purchases may instead be related to informality, which is prevalent in Peru. Specifically, by formalizing more transactions, e-invoicing allowed firms to claim deductions on previously unreported purchases. This is consistent with purchases increasing more among small firms, where informality and misreporting issues tend to be larger, as we will see below.

**Table 1:** Impact of mandatory e-Invoicing.

	(1)	(2)	(3)	(4)	(5)	(6)	(7)	(8)
	Taxable	Taxable	VAT	New VAT	VAT	Share of	Employed	Sales per
	sales	purchases	liabilities	credits	payments	taxable VA	workers	worker
Treatment	0.0743∗∗∗	0.0549∗∗∗	0.0820∗∗∗	0.0477	0.0544	−0.00216	0.0163∗∗∗	0.0439∗∗∗
(first year)	(0.0133)	(0.0146)	(0.0215)	(0.0390)	(0.0337)	(0.00522)	(0.00473)	(0.0106)
								
Observations	1,010,380	1,010,380	1,010,380	1,010,380	1,010,380	1,010,380	936,527	936,527

**Note:** Results for the balanced sample of firms mandated to adopt e-invoicing before 2019. The last two columns drop firms with no reported workers. The inverse hyperbolic sine transformation is applied to all dependent variables, which are originally measured in constant 2014 Peruvian soles. The share of taxable VA is the ratio of taxable to total value added. The treatment indicator is equal to one in the quarter of mandatory e-invoicing adoption and the following four quarters. All specifications include quarter fixed effects, firm fixed effects, firm-specific linear trends and a variable controlling for the fifth and following quarters after the date of mandatory e-invoicing adoption. Table A.5 shows estimates with the additional controls. Firm-clustered standard errors are shown in brackets. ∗ 0.10, ∗∗ 0.05, ∗∗∗ 0.01.

Next, we look at the impact of e-invoicing on VAT liabilities and new VAT credits. Taxable sales and purchases result in VAT liabilities when taxable value added is positive (i.e. sales exceed purchases), or new VAT credits when it is not. We construct these two variables accordingly, setting VAT liabilities (credits) equal to zero when taxable sales are lower (higher) than taxable purchases. The vast majority of observations (83 percent) exhibit positive taxable value added, and the estimated 8.2 percent significant increase in VAT liabilities is therefore consistent with the increase in both sales and purchases. We also estimate a positive and smaller increase in new VA credits, although insignificant, reflecting the response of some firms with negative taxable value added.

The next column in Table 1 examines the impact of e-invoicing on VAT payments. These represent the actual VAT collected by the tax authority after accounting for VAT credits, tax arrears and other offsets. Except for export-oriented firms, Peruvian firms cannot obtain cash refunds from VAT credits but can accumulate these credits to offset future VAT liabilities. Therefore, a firm’s VAT payments do not necessarily correspond to the amount of newly incurred VAT liabilities as past VAT credits are used to reduce how much they owe. Indeed, we find that VAT payments are estimated to increase by 5.4 percent on average, and at a lower rate compared with VAT liabilities, either because of tax offsets or because of payment non-compliance. The coefficient estimate is also not statistically significant.

We explore in the column six whether the impact of e-invoicing is driven by a re-classification of tax-exempt sales or purchases. This would happen if firms began reporting as taxable certain transactions that they had previously reported as nontaxable. We reject this hypothesis as we estimate the change in the ratio of taxable value added to total value added to be small and insignificant.

**Fig. 4:**
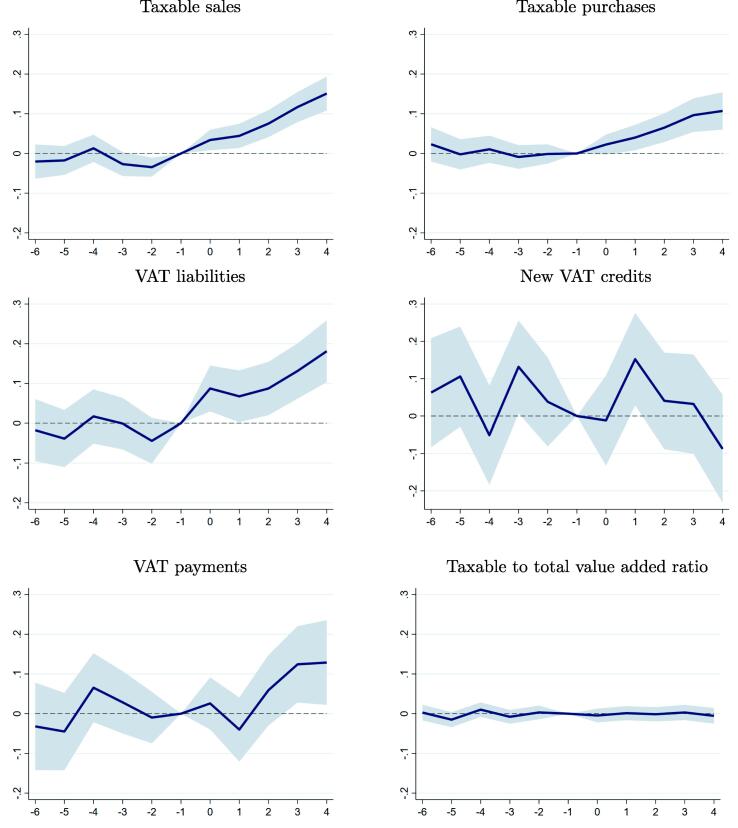
Impact of e-Invoicing Around the Mandatory Date of Adoption. **Note:** This figure plots the point estimates (solid line) and the 95 percent confidence intervals (shaded area) of the $\beta_{t}$ coefficients in Eq. (2), estimated using the balanced sample of firms mandated to adopt e-invoicing before 2019. The responses represent the percent change in the dependent variable relative to the mandatory adoption quarter.

In Fig. 4, we confirm our findings using the dynamic panel specification in Eq. 2, which is estimated using the same balanced sample. This specification allows us to rule out the presence of differential trends between the treated and control firms before the e-invoicing reform, as in general the estimated coefficients in the six quarters prior to the mandated date of adoption are not significantly different from zero. The small dip in taxable sales two quarters before treatment is likely due to sample noise, since we do not observe similar trends in other variables like taxable purchases or VAT liabilities, and there is no clear incentive for firms to increase sales under-reporting before the deadline.

Following the introduction of e-invoicing, we find that taxable sales and purchases rise steadily, in line with the gradual increase in actual e-invoicing adoption rates documented in Fig. 2 after the original deadline dates, and possibly also reflecting some initial adjustment costs that could temporarily divert resources away from production. Four quarters after the mandatory date of adoption, e-invoicing is associated with an average increase of 15 percent in taxable sales and 11 percent in taxable purchases. These effects are larger than the estimated effects shown in Table 1 because those results represent the average impact over the entire first year of e-invoicing adoption, and not just the fourth quarter. As shown in the middle panel, the response of reported VAT liabilities mirrors the impact on taxable sales and purchases, while changes in new VAT credits do not reveal a particular trend and are mostly insignificant.

In the bottom left panel, we see that actual VAT payments also move up gradually, with a statistically significant increase of 13 percent in the fourth quarter after e-invoicing was made mandatory. The somewhat different paths for VAT liabilities and payments mostly reflects the fact that firms can (and did) use VAT credits to lower their tax liability. It is also possible that the transition to e-invoicing may have interacted in complex ways with Peru’s special VAT withholding regimes, which require that certain taxpayers withhold part of the VAT liability of their suppliers or customers, breaking the link between liabilities and actual payments. As seen in the bottom right panel, there is also no change in ratio of taxable value added to total value added in the period around the e-invoicing reform.

**Fig. A.3:**
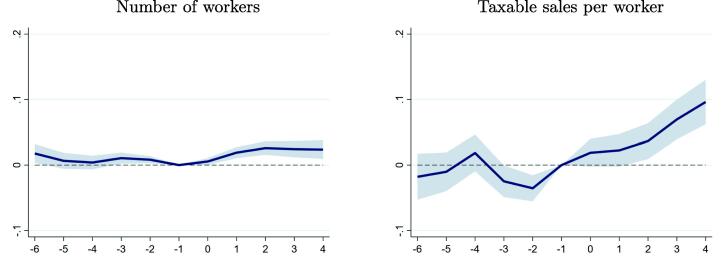
Impact of e-Invoicing Around the Mandatory Date of Adoption. **Note:** This figure plots the point estimates (solid line) and the 95 percent confidence intervals (shaded area) of the $\beta_{t}$ coefficients in Eq. (2), estimated using the balanced sample of firms mandated to adopt e-invoicing before 2019. The responses represent the percent change in the dependent variable relative to the mandatory adoption quarter.

The two last columns in Table 1 examine the effect of e-invoicing on the number of workers employed by firms and on sales per worker (see also Fig. A.3 in the appendix).^21^ The employment data is drawn from other tax records, and only captures formal workers. We find that the e-invoicing reform had a positive impact on firm employment, which is estimated to increase 1.6 percent in the first year of the reform. Taxable sales per worker also rises in the first year following the adoption requirement, with an average increase of 4.4 percent. Taken together, these results show that the e-invoicing reform was associated with higher reported economic activity, and that this was achieved without a substantial change in labor input. The increase in firm employment also suggests that e-invoicing may have some real effects on firm behavior, in addition to improved tax compliance and reporting. However, data limitations mean that we cannot fully separate the relative importance of these compliance and productivity changes.

All these results are estimated using the balanced sample of firms with positive sales throughout the estimation period. We also estimate treatment effects on the full sample including entering and exiting firms, which are reported in the appendix in Table A.7 Panel F. As shown in the summary statistics (Appendix Table A.1, Table A.3), these firms tend to be smaller and pay less VAT. The estimated impacts are larger when looking at the full sample, both because the treatment is larger for these smaller firms and because of composition effects induced by the post-treatment exit of smaller firms.

### 5.2 Robustness tests

#### 5.2.1 Impact of early E-invoicing adopters

We implement a number of robustness checks confirming our baseline results. First, some firms adopted e-invoicing before the mandatory deadline, which could bias our treatment effect estimator. In order to parse out how these early adopters and voluntary compliers may be influencing our result we conduct robustness checks where these groups are excluded from the analysis sample. Table 2 Panel A shows the results for the baseline specification using the subsample of firms which excludes both early adopters from the treated firms and voluntary adopters from the control firms. Our results are essentially unchanged, suggesting no systemic bias from voluntary or early adopters.

**Table 2:** Impact of mandatory e-Invoicing - robustness.

	(1)	(2)	(3)	(4)	(5)	(6)	(7)	(8)
	Taxable	Taxable	VAT	New VAT	VAT	Share of	Employed	Sales per
	sales	purchases	liabilities	credits	payments	taxable VA	workers	worker
**A. Excluding firms that adopted e-invoicing before adoption deadlines**								
Treatment	0.0803∗∗∗	0.0585∗∗∗	0.0818∗∗∗	0.0619	0.0528	−0.00383	0.0130∗∗	0.0501∗∗∗
(first year)	(0.0152)	(0.0168)	(0.0241)	(0.0435)	(0.0378)	(0.00585)	(0.00529)	(0.0122)
								
Observations	880,270	880,270	880,270	880,270	880,270	880,270	812,486	812,486
**B. Only final consumer-oriented sectors**								
Treatment	0.0470∗	0.0254	0.136∗∗∗	0.121∗	0.119∗∗	−0.00303	0.00943	0.0327
(first year)	(0.0275)	(0.0312)	(0.0421)	(0.0719)	(0.0592)	(0.0110)	(0.00781)	(0.0210)
								
Observations	243,337	243,337	243337	243,337	243,337	243,337	221,915	221,915

**Note:** These are results for the balanced sample of firms mandated to adopt e-invoicing before 2019. The last two columns drop firms with no reported workers. The inverse hyperbolic sine transformation is applied to all dependent variables, which are originally measured in constant 2014 Peruvian soles. The share of taxable VA is the ratio of taxable to total value added. The treatment indicator is equal to one in the quarter of mandatory e-invoicing adoption and the following four quarters. All specifications include quarter fixed effects, firm fixed effects, firm-specific linear trends and a variable controlling for the fifth and following quarters after the date of mandatory e-invoicing adoption. Table A.8 shows estimates with additional controls. Firm-clustered standard errors are shown in brackets. ∗ 0.10, ∗∗ 0.05, ∗∗∗ 0.01.

#### 5.2.2 Firm spillovers

Next, we tackle concerns about spillovers between connected treatment and control firms. For example, our results on taxable sales could be biased upward if control firms lower their sales in response to the reform. This could happen if treated firms that purchase from control firms decide to lower or terminate these purchases because those suppliers have not yet adopted e-invoicing. To address this, we limit the estimation sample to firms drawn from sectors that primarily sell to final consumers (households or the government), where these concerns about spillovers are lessened.

Using the latest available input–output (IO) table for Peru (2015), we identify the following industry classifications as making more than 80 percent of their domestic sales to final consumers: Hotels/Restaurants, Health, Public Administration, Food, Drinks and Alcohol, Education and Telecommunications. The IO table combines wholesale and retail trade under the same classification, and therefore this classification does not meet the 80 percent threshold in aggregate. Industry codes from our database, however, allow us to further distinguish firms engaged in wholesale trade from those engaged in retail trade. We are consequently able to add firms engaged in retail trade to our control group estimation sample, while excluding wholesale trade firms.

Though the size of the estimation sample falls significantly, resulting in attenuation of the estimates and larger standard errors, our results in Table 2 Panel B are qualitatively similar to the original specification, with increases in sales, VAT liabilities and VAT payments captured in the year following the reform. This would provide support for the argument that the treatment effect is not being driven by inter-firm spillovers. In appendix Table A.8 Panel J we use a broader sectoral aggregation in order to supplement sample size. Hence, we include sectors which make more than 60 percent of their domestic sales to final consumers. The results are again consistent with our baseline and the coefficients are estimated with greater precision than the stricter threshold of 80 percent. We would argue, therefore, that spillovers are not systematically biasing us towards finding a treatment effect.

#### 5.2.3 Firms engaged in fraud

Next, we examine the robustness of our results to including firms that were caught issuing or claiming fraudulent invoices. While one would expect that these firms would respond more strongly to the e-invoicing reform, in practice a large majority becomes inactive after being caught, and many of those that remain open have infrequent (non-fraudulent) sales. As a result, there are only about 600 such firms captured in our baseline sample. Moreover, these firms transitioned to e-invoicing at lower rates than other firms, as seen in Fig. A.2 in the appendix. Overall, it is hard to identify the impact of e-invoicing, and to identify it separately from the impact of tax audits, increased monitoring and more frequent enforcement activities. While we leave these firms out of the baseline specification, our results are similar if we include these additional firms (Appendix Table A.5 Panel B).

#### 5.2.4 Alternative controls

Finally, we also find that our results remain essentially unchanged when we use a more parsimonious specification with wave trends instead of firm-specific trends (appendix Table A.5 Panel C), or if we additionally control for firm employment, wage bill and its capital stock (appendix Table A.6 Panel E) in our baseline specification.

### 5.3 Heterogeneity by firm size, creditor status, and sector

#### 5.3.1 Firm size

We next assess the potential heterogeneity in treatment effects, starting by examining the role of firm size. Much of the existing research on the impact of tax audits finds larger treatment effects among smaller firms (e.g., Kleven, 2016). Small firms are less likely to be subject to tax audits, since individually they make only a marginal contribution to overall tax collections. They also tend to conduct more transactions in cash, which makes them more difficult to record and track. Smaller firms are thus more likely to engage in tax noncompliance, and an increase in the threat of audit should disproportionately affect their behavior (Slemrod, 2019).

**Fig. 5:**
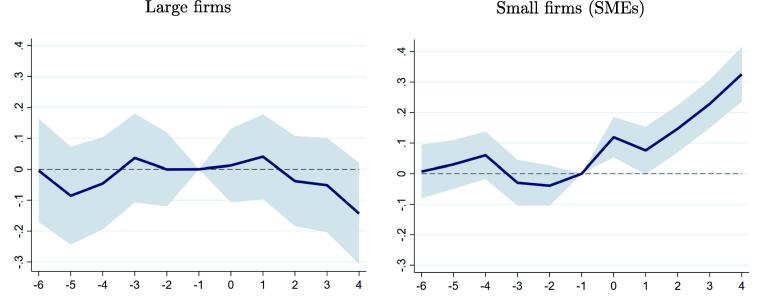
Impact of e-Invoicing on VAT liabilities by Firm Size. **Note:** This figure plots the point estimates (solid line) and the 95 percent confidence intervals (shaded area) of the $\beta_{t}$ coefficients in Eq. (2), estimated using the balanced sample of firms mandated to adopt e-invoicing before 2019. Small and medium enterprises are defined as having sales lower than 2,300 UIT (about $3 million) in 2013Q2. The responses represent the percent change in the dependent variable relative to the mandatory adoption quarter.

We start by re-estimating our dynamic difference-in-differences specification in Eq. 2, but this time focusing on the response of VAT liabilities separately for small and large firms. We define small firms as those having annual taxable sales below 2,300 UIT (about $3 million) at the beginning of our sample period, as this threshold is consistent with the legal definition of a small or medium firm in the Peruvian legal system. As Fig. 5 makes clear, the significant increase in VAT liabilities following e-invoicing is entirely driven by the response of SMEs, whereas larger firms are essentially unaffected.

In Table 3, we formally test the hypothesis that e-invoicing had a different impact on small and medium firms relative to large firms by interacting the treatment indicator with an indicator of firm size. As the first columns show, the increases in taxable sales and purchases, VAT liabilities and actual VAT payments are mostly concentrated among smaller firms, and the firm size interaction coefficients are statistically different from zero. E-invoicing had a much smaller impact on taxable sales for large firms, which suggests that under-reported sales were more prevalent among small firms. In addition, it had essentially no impact on taxable purchases reported by large firms, which is consistent with anecdotal evidence that fake invoices were not prevalent among large firms, and with the idea that smaller firms increased reported purchases because e-invoicing formalized previously unreported transactions. Interestingly, while e-invoicing had a positive but not statistically significant impact on gross VAT liabilities for large firms, it appears to have been associated with a decline in actual VAT payments, as calculated after VAT credits and other offsets were applied. However, this impact appears to be driven by differences in VAT credit status, as discussed below. These results are robust to using firm sales at the beginning of the sample as an alternative measure of firm size, as we report in appendix Table A.11.

**Table 3:** Heterogeneity by firm size and creditor status.

	By Firm Size				By Firm Size and Creditor Status			
	Taxable	Taxable	VAT	VAT	Taxable	Taxable	VAT	VAT
	sales	purchases	liabilities	payments	sales	purchases	liabilities	payments
**Treatment (first year) interacted with**								
× SME dummy	0.106∗∗∗	0.101∗∗∗	0.116∗∗∗	0.196∗∗∗				
	(0.0183)	(0.0197)	(0.0251)	(0.0465)				
								
× large firm dummy	0.0379∗∗	0.00114	0.0432	−0.109∗∗				
	(0.0183)	(0.0204)	(0.0339)	(0.0453)				
**Treatment (first year) interacted with**								
× SME dummy ×					0.122∗∗∗	0.0940∗∗∗	0.166∗∗∗	0.343∗∗∗
no VAT credit dummy					(0.0201)	(0.0217)	(0.0249)	(0.0492)
								
× SME dummy ×					0.0611	0.123∗∗∗	−0.0278	−0.229∗∗
VAT credit dummy					(0.0393)	(0.0420)	(0.0629)	(0.105)
								
× large firm dummy ×					0.0626∗∗∗	0.0104	0.0844∗∗∗	0.00277
no VAT credit dummy					(0.0214)	(0.0230)	(0.0302)	(0.0504)
								
× large firm dummy ×					−0.0158	−0.0194	−0.0457	−0.353∗∗∗
VAT credit dummy					(0.0343)	(0.0406)	(0.0839)	(0.0907)
								
Observations	1,010,380	1,010,380	1,010,380	1,010,380	1,010,380	1,010,380	1,010,380	1,010,380

**Note:** Results for the balanced sample of firms mandated to adopt e-invoicing before 2019. The inverse hyperbolic sine transformation is applied to all dependent variables, which are originally measured in constant 2014 Peruvian soles. The treatment indicator is equal to one in the quarter of mandatory e-invoicing adoption and the following four quarters. This treatment indicator is interacted with two dummy variables indicating if a firm had sales lower than 2,300 UIT (about $3 million) or a positive stock of VAT credits in 2013Q2, respectively. All specifications include quarter fixed effects, firm-specific trends and controls for the effects in the fifth and following quarters after the date of mandatory e-invoicing adoption. Appendix Table A.9, Table A.10 additionally shows estimates with these controls. Firm-clustered standard errors are shown in brackets. ∗ 0.10, ∗∗ 0.05, ∗∗∗ 0.01.

#### 5.3.2 VAT credit status

In the remaining columns of Table 3, we examine the role played by the stock of VAT credits firms have, which is a potential confounder in the relation between e-invoicing and firm size. About one-third of the firms in our sample carried a stock of VAT credits at the beginning of our sample period, which they could use to offset VAT liabilities (see Table A.4 in appendix). To understand how these outstanding VAT credits might affect the impact of the e-invoicing reform, we interact the treatment variable with an indicator for whether firms had a positive stock of VAT credits at the beginning of the sample period. The results are shown in last four columns of Table 3, where once again we find large and significant differences across firms with and without past credits. Firms without VAT credits experience a stronger increase in VAT liabilities, accumulate fewer new VAT credits and pay more VAT after e-invoicing was made mandatory. By contrast, firms with existing VAT credits not only avoided paying additional VAT after e-invoicing was introduced, but also managed to accumulate new credits, essentially offsetting the positive impact of e-invoicing on VAT liabilities and payments.

#### 5.3.3 Economic sector

Next, we evaluate how different economic sectors responded to the e-invoicing reform. We focus on the impact on the reform on six main sectors of the Peruvian economy, namely construction, manufacturing, transportation, trade, hospitality and business and professional services.^22^. We start by examining the dynamics of taxable sales and purchases across sectors in Table 4 (see also Fig. A.6 in the appendix). Following the reform, we observe a large and significant increase of 24.4 percent in reported taxable sales in the construction sector in the first year, and smaller though still significant increases in transportation, trade and professional and business services ranging between 4 to 8 percent. Taxable purchases follow a similar pattern but increase less than taxable sales. The overall impact on construction and services is particularly noticeable, since these sectors were previously identified as having large VAT compliance gaps in Peru, which suggests that mandatory e-invoicing may induce changes for firms with low compliance (Keen, 2013, IMF, 2015). However, we see little impact of e-invoicing in the hospitality sector.

**Table 4:** Heterogeneity by economic sector.

	Taxable	Taxable	VAT	New VAT	VAT
	sales	purchases	liabilities	credits	payments
Construction	0.244∗∗∗	0.167∗∗	0.0759	−0.00292	0.227
	(0.0664)	(0.0682)	(0.133)	(0.208)	(0.168)
					
Manufacturing	0.0526∗	0.0555∗∗	0.110	−0.0809	0.260∗∗∗
	(0.0298)	(0.0251)	(0.0709)	(0.130)	(0.0827)
					
Transportation	0.0774∗∗	0.0247	0.160∗∗∗	−0.0212	0.187
	(0.0371)	(0.0414)	(0.0566)	(0.122)	(0.122)
					
Trade	0.0456∗∗	0.0404	0.0300	0.244∗∗∗	−0.243∗∗∗
	(0.0224)	(0.0248)	(0.0337)	(0.0691)	(0.0624)
					
Hotels and Restaurants	−0.00160	0.0375	0.144	0.182	0.112
	(0.0289)	(0.0312)	(0.115)	(0.141)	(0.106)
					
Services	0.0633∗∗∗	0.0587∗∗	0.0921∗∗	−0.159∗∗	0.191∗∗∗
	(0.0238)	(0.0288)	(0.0380)	(0.0708)	(0.0535)
Controls	Yes	Yes	Yes	Yes	Yes

**Note:** This table presents estimated treatment effect for separate difference-in-difference regressions for different economic sectors. All regressions use the balanced sample of firms mandated to adopt e-invoicing before 2019. The inverse hyperbolic sine transformation is applied to all dependent variables, which are originally measured in constant 2014 Peruvian soles. The treatment indicator is equal to one in the quarter of mandatory e-invoicing adoption and the following four quarters. All specifications include quarter fixed effects, firm-specific trends and a variable controlling for the fifth and following quarters after the date of mandatory e-invoicing adoption. Firm-clustered standard errors are shown in brackets. ∗ 0.10, ∗∗ 0.05, ∗∗∗ 0.01.

Turning to VAT liabilities and payments, it is instructive to contrast the response of firms in the trade and business and professional services sectors, as there seem to be striking differences in the impact of e-invoicing across the two sectors. In the case of the trade sector, we find a strong and significant increase in new VAT credits, whereas the new VAT credits decline in the services sector after e-invoicing was introduced. As a result, we find a significant increase in VAT payments in the services sector, but an average decline in VAT payments among firms in the trade sector. The positioning of these firms in their value chains could offer some explanation for this pattern. Firms providing professional and business services are more often upstream industries that do not require many inputs from other sectors. Conversely, the trade sector requires sourcing from many industries and selling to a large extent to final consumers. Therefore, firms in the trade sector are more likely to accumulate large stocks of VAT credits that they could use to offset their VAT liabilities.

### 5.4 Anticipation effects and firm survival

In this section, we examine anticipation effects after e-invoicing adoption requirements were announced, but before adoption deadlines were reached. We do not expect anticipation effects for reported transactions and VAT variables before the e-invoicing adoption deadlines. Treated firms have no reasons to expect a change in monitoring from the tax authorities before they adopt e-invoicing, and as such have no incentive to change their reporting and VAT compliance. However, if e-invoicing reduces noncompliance, thereby increasing the effective tax rate on firms, one would expect some firms to exit if they cannot maintain profitability under a higher effective tax burden. We would also expect exits if e-invoicing adoption costs and the expected costs of non-adoption are large for some firms. Further, we expect a treated firm to exit before or at the adoption deadline, that is before its fails to adopt e-invoicing or before the additional scrutiny resulting from e-invoicing adoption exposes the firm as non-compliant. We test these hypotheses by examining changes in firm reporting, compliance and survival around both the adoption deadlines and the announcement dates.

**Fig. 6:**
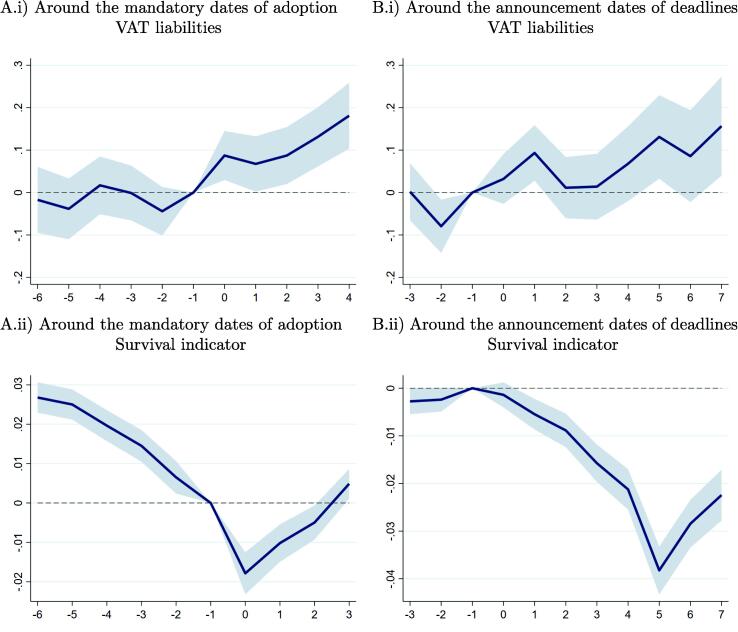
e-Invoicing Impact Around Announcement Dates and Adoption Deadlines. **Note:** In the (A) panels, the reference period −1 corresponds to the quarter before the deadline for adopting e-invoicing. In (B) panels, instead of using the deadlines for adoption as before, the graph shows the relatives changes of the treated around the *announcement dates*, that is when deadlines were announced. In top panels (i), results are estimated using the 2013Q2–2017Q4 balanced sample of all firms mandated to adopt e-invoicing before 2019. In bottom panels (ii), the dependent variable is a firm indicator of survival that takes the value one until the last quarter where positive sales are observed and the value zero from then on to the end of the sample; the estimation sample excludes the last quarter (2017Q4) because survival cannot be computed then. This figure plots the point estimates (solid line) and the 95 percent confidence intervals (shaded area) of the $\beta_{t}$ coefficients in Eq. (2) .

**Fig. A.4:**
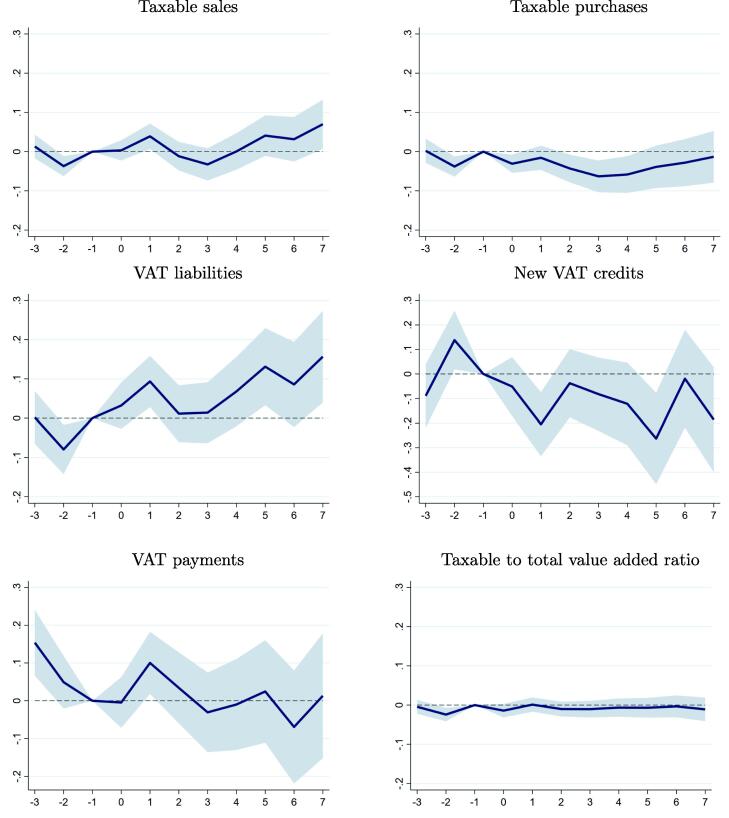
Impact of e-Invoicing Around the Announcement Dates. **Note:** Instead of using the deadlines for adoption as in the main text, these graphs show the relatives changes of the treated around the *announcement dates*, that is when deadlines were announced. This figure plots the point estimates (solid line) and the 95 percent confidence intervals (shaded area) of the $\beta_{t}$ coefficients in Eq. (2), estimated using the balanced sample of firms mandated to adopt e-invoicing before 2019. The responses represent the percent change in the dependent variable relative to the announcement quarter (quarter 0 on the horizontal axis).

We find no evidence of anticipation effects for VAT liabilities. The upper left panel in Fig. 6 reproduces the changes in VAT liabilities around the adoption deadlines from Fig. 4 and displays a clear inflection at the adoption deadline. In the upper right panels, we use again Eq. 2, but alternatively set the reference period as the quarter before the deadline was announced. By contrast with previous results around the adoption deadlines, this panel shows no clear signs of change in VAT liabilities around the announcement dates. Fig. A.4 in the appendix confirm this pattern for taxable sales, taxable purchases, and VAT payments. The coefficient estimates of the average treatment effects after the announcement dates in Table A.7 Panel G in the appendix are also mostly insignificant and attenuated compared to treatment estimates following the adoption deadlines.

Next, we examine extensive margin responses. We define survival as having positive sales in any of the observed future quarters. The new dummy variable equals one until the quarter before the last observed sales and zero from then on to the end of the sample period.

The announcement of future e-invoicing requirements has a significant impact on firm survival. The bottom left panel in Fig. 6 shows that differences in firm survival pre-exist before the adoption deadlines. When considering survival around the announcement dates in the bottom right panel, pre-treatment differences are insignificant, and firm survival starts to decline significantly only after the announcement dates. We also find that firm survival reaches its minimum about a year after the announcement dates, and that this corresponds to adoption deadlines. Consistent with our hypothesis, the results suggest that some firms stopped reporting to the tax authorities or ended activity altogether in response to the announcements. This behavior would be consistent with non-compliant firms expecting increased scrutiny from the tax authorities once they start e-invoicing or with fixed e-invoicing adoption costs that are too high for some firms.

In appendix Fig. A.7, we also examine heterogeneity in survival around announcement dates between different sectors. Once again, the sectors that were previously identified as having large VAT compliance gaps in Peru firms features the strongest response. Firms in construction, in services, in retail and, to some extent, in manufacturing have the largest and most significant drops in survival probability in the quarters following the announcement dates.

**Fig. A.5:**
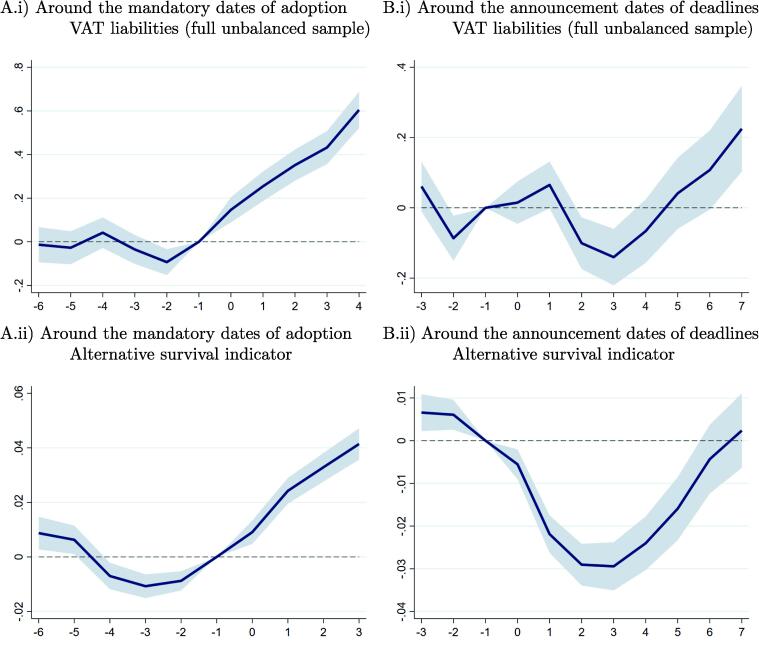
E-Invoicing Impact Around Announcement Dates and Adoption Deadlines. **Note:** In the (A) panels, the reference period −1 corresponds to the quarter before the deadline for adopting e-invoicing. In (B) panels, instead of using the deadlines for adoption as before, the graph shows the relatives changes of the treated around the *announcement dates*, that is when deadlines were announced. In top panels (i), results are estimated using the 2013Q2–2017Q4 unbalanced sample of all firms mandated to adopt e-invoicing before 2019, including firms that enter and exit during the estimation period. In bottom panels (ii), the dependent variable is a firm indicator of survival that takes the value one if positive sales are observed next quarter and zero otherwise; the estimation sample excludes the last quarter (2017Q4) because survival cannot be computed then. This figure plots the point estimates (solid line) and the 95 percent confidence intervals (shaded area) of the $\beta_{t}$ coefficients in Eq. (2).

**Fig. A.6:**
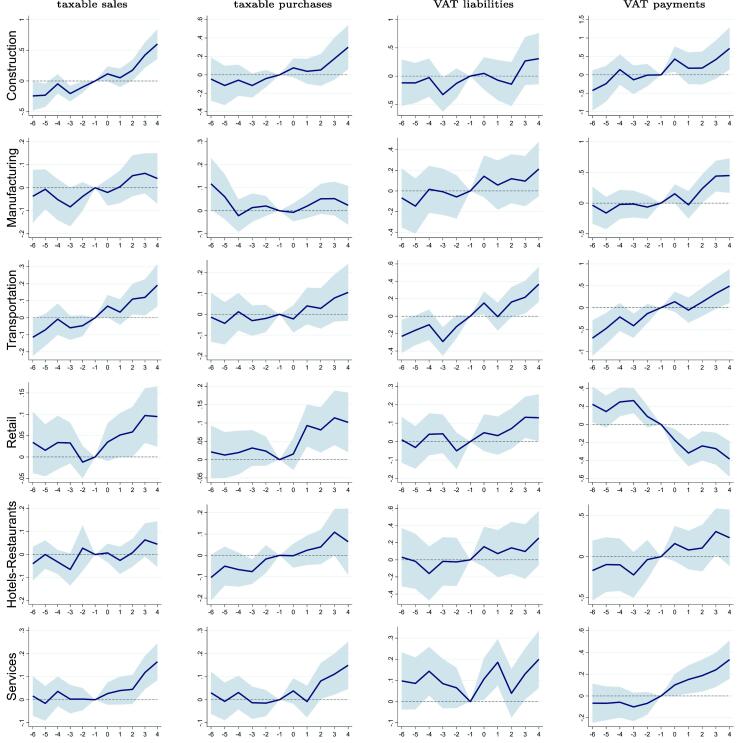
Impact of e-Invoicing on VAT liabilities by Sector. **Note:** This figure plots the point estimates (solid line) and the 95 percent confidence intervals (shaded area) of the $\beta_{t}$ coefficients in Eq. (2), estimated using the balanced sample of firms mandated to adopt e-invoicing before 2019, excluding waves where firms were caught in fraudulent transactions. Each panel corresponds to a separate regression by sector. The responses represent the percent change in the dependent variable relative to the mandatory adoption quarter.

**Fig. A.7:**
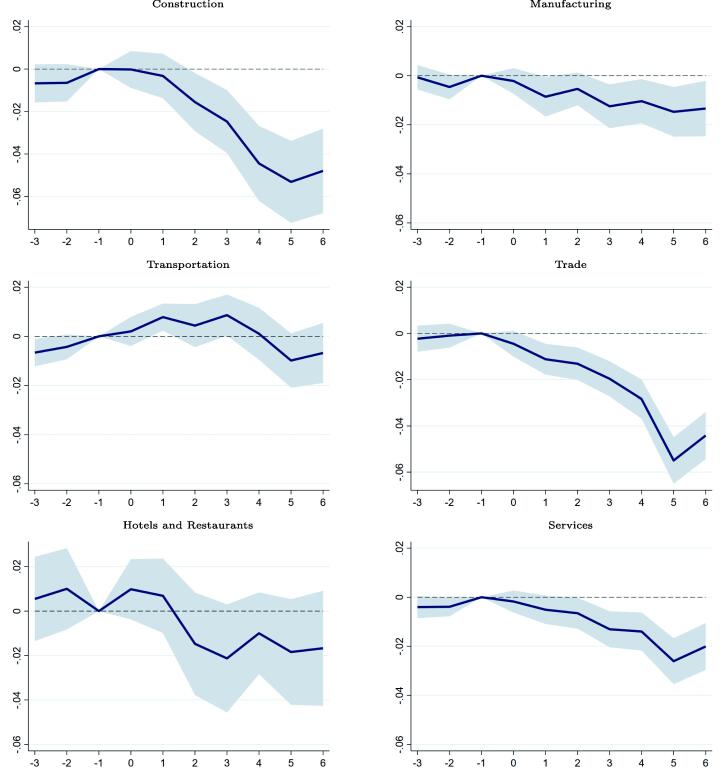
Impact of e-Invoicing on Survival Rates by Sector. **Note:** Instead of using the deadlines for adoption as in the main text, these graphs show the relatives changes of the treated around the *announcement dates*, that is when deadlines were announced. The dependent variable is a firm indicator that takes the value zero until the last quarter where positive sales are observed and the value one from then on to the end of the sample. The last quarter (2017Q4) is dropped. This figure plots the point estimates (solid line) and the 95 percent confidence intervals (shaded area) of the $\beta_{t}$ coefficients in Eq. (2), estimated using the sample of all firms mandated to adopt e-invoicing before 2019. Each panel corresponds to a separate regression by sector.

We also examined the robustness of our results by considering an alternative definition of survival to address concerns related to the interpretation of results towards the end of the sample. Concerns arise because the closer we are to the end of the sample, the fewer future periods we observe, and the less likely the variable is to correctly identify survival for firms that sell intermittently. Therefore, we define an alternative firm survival variable that is equal to one if we observe positive sales at the firm in the following quarter, and zero otherwise. Result in Fig. A.5 in appendix are qualitatively similar. In the same figure, we also report the changes in VAT liabilities in the full unbalanced sample, first around the adoption deadline, and then around the announcement dates. Our conclusions are robust to the use of this broader sample.

### 5.5 IV estimation of the effects of e-Invoicing adoption

The results shown in the previous sections focus on the impact of directing firms to adopt e-invoicing. However, some firms delayed adoption for a few quarters, and others kept using paper invoices for more than a year after the deadline had passed. Therefore, the magnitude of these effects may be different from the effect of actually adopting e-invoicing. In Table 5, we estimate the effects of actual adoption by comparing mandated adopters with non-mandated non-adopters using the instrumental variable approach described in Eqs. (3), (4).

**Table 5:** Instrumental variable results.

	1st Stage	2nd Stage				
	Adoption	Taxable	Taxable	VAT	New VAT	VAT
		sales	purchases	liabilities	credits	payments
Treatment	0.448∗∗∗					
(first year)	(0.00396)					
						
Adoption		0.166∗∗∗	0.123∗∗∗	0.183∗∗∗	0.106	0.121
		(0.0297)	(0.0325)	(0.0480)	(0.0872)	(0.0753)
Robust F-stat	1.30E+04					
Observations	1,010,380	1,010,380	1,010,380	1,010,380	1,010,380	1,010,380

**Note:** Regression results for the balanced sample of firms mandated to adopt e-invoicing before 2019. The inverse hyperbolic sine transformation is applied to all dependent variables, which are originally measured in constant 2014 Peruvian soles. The treatment indicator is equal to one in the quarter of mandatory e-invoicing adoption and the following four quarters. All specifications control include quarter fixed effects, firm-specific trends and a variable controlling for the fifth and following quarters after the date of mandatory e-invoicing adoption. Appendix Table A.12 additionally shows estimates for controls. Firm-clustered standard errors are shown in brackets. ∗ 0.10, ∗∗ 0.05, ∗∗∗ 0.01.

The first column of Table 5 shows that assignment to treatment strongly predicts actual adoption across all treatment groups. The Kleibergen-Paap F-statistic for the first-stage regression is very large, indicating that there is no concern about weak instruments. Moreover, we argue that the exclusion restriction on our instrument is met because we find no consistent evidence of anticipation effects on a range of outcome variables in our dynamic specification and the treatment effect is coincident with the date of the reform, with no effects being found at the date of the announcement of e-invoicing requirements.

We find that adopting e-invoicing is associated with statistically significant increases in taxable sales and purchases of 16.6 and 12.3 percent, respectively, and also an 18.3 percent increase in reported VAT liabilities. The impact on new VAT credits and actual VAT payments is also positive, as before, although not statistically significant. In the case of payments, this is likely driven by the differences in e-invoicing impact by VAT credit status, as discussed above. Still, the larger magnitude of these impacts suggests our ITT estimates are smaller due to noncompliance among the mandated firms, or voluntary compliance among firms in the control group, and that the effects of the reform on firms that have indeed adopted e-invoicing are substantially larger.

## 6 Conclusion

This paper investigates the effect of e-invoicing adoption on firm performance and tax compliance using administrative tax data on all VAT paying firms in Peru. We show that e-invoicing increases reported firm sales, purchases and VAT liabilities by over 5 percent on average in the first year after adoption. These effects are heterogeneous across firms, with larger impacts for small firms and firms in sectors with a higher risk of tax noncompliance. In addition, we find that the announcement of the e-invoicing reform is associated with a temporary decline in the firm survival rate, particularly in higher risk sectors. Together, this suggests that the impact of e-invoicing is operating primarily through the deterrence channel of reduced noncompliance.

Applying our regression estimates from Table 3, a back-of-the-envelope calculation suggests that e-invoicing increased total gross VAT liabilities by 6.7 percent in the first year of the reform, driven by higher liabilities among SMEs and large firms that have no stocks of VAT credits. However, total VAT payments declined by 3.1 percent, as a significant number of both small and large firms relied on existing VAT credits to offset their higher VAT liabilities. While this suggests that e-invoicing should eventually lead to an increase in VAT collections, that revenue impact may materialize slowly given the very large stock of existing credits in the Peruvian VAT system. Nevertheless, we should emphasize that the e-invoicing reform had a significant compliance impact among smaller firms, who are traditionally difficult and costly to monitor through conventional audit techniques and are therefore typically not subject to extensive monitoring or audit.

The effects of e-invoicing build up gradually over time, implying that the full effect of the reform is not yet fully accounted for. Moreover, by the end of our sample period, the tax authority had not yet made significant changes to its risk management strategy to make use of the flow of information generated by the e-invoicing system. Therefore, our results identify changes in firm behavior in response to a perceived increase in the threat of audit, which is likely a lower bound for the full effect of an e-invoicing reform once improved monitoring and enforcement are in place based on this new technology.

We also find that e-invoicing adoption appears to have had a stronger impact on VAT collections in upstream industries. A useful avenue for future research would be to study the spillover effects of e-invoicing adoption on upstream and downstream firms, as digitalization in some firms can strengthen the incentives for connected firms to also digitalize and improve compliance (e.g., Keen and Lockwood, 2010, Pomeranz, 2015).

## Declaration of Competing Interest

The authors declare that they have no known competing financial interests or personal relationships that could have appeared to influence the work reported in this paper.

## Figures and Tables

**Table A.1:** Full sample summary statistics over 2014–2017: main variables.

**Note:** Waves correspond to groups of firms and are identified by initial deadline for e-invoicing adoption. Adoption deadlines are indicated in brackets at the column tops. Values correspond to annual averages and are expressed in thousands of 2014 soles, unless otherwise noted. In 2014, the exchange rate was approximately 0.34 US$ per soles. Shaded columns indicate the waves that are excluded from the main text estimation sample, and capture firms that were caught in fraudulent transactions before their assignment to a wave (waves 2 and 6) and firms not mandated into e-invoicing by end 2017. ∗ indicates that numbers correspond to the share of invoices issued electronically. ∗∗ indicates that numbers correspond to a dummy variables indicating if a firm exports.

**Table A.2:** Full sample summary statistics over 2014–2017: group distributions.

**Note:** For every wave, the first column shows the percentage of observations that belongs to the corresponding industry, size or VAT credit group. Value added refers to annual averages and is expressed in thousands of 2014 soles. In 2014, the exchange rate was approximately 0.34 US$ per soles. Shaded columns indicate the waves that are excluded from the main text estimation sample, and capture firms that were caught in fraudulent transactions before their assignment to a wave (waves 2 and 6) and firms not mandated into e-invoicing by end 2017. ∗ SMEs are firms with inflation-adjusted tax units between 150 and 2300, i.e. 0.6 and 8.7 million soles in 2014. ∗∗ refers to firms with a positive stock of VAT credits in 2013Q2.

**Table A.3:** Balanced sample summary statistics over 2014–2017: main variables.

	Wave 1		Wave 3		Wave 4		Wave 5		Wave 7	
Values in	(October 2014)		(July 2015)		(July 2016)		(December 2016)		(many 2018 deadlines)	
thousand 2014 soles	mean	sd	mean	sd	mean	sd	mean	sd	mean	sd
total sales	1,321,000	(1,763,000)	169,000	(183,700)	102,300	(218,900)	18,860	(138,900)	2,896	(8,679)
total purchases	1,053,000	(1,743,000)	121,700	(144,800)	83,570	(144,600)	13,760	(92,770)	2,271	(7,567)
value added	364,200	(748,700)	56,720	(112,300)	29,210	(149,800)	6,286	(76,440)	806	(4,169)
gross VAT	165,000	(242,900)	23,570	(23,460)	14,510	(20,910)	2,060	(7,426)	413	(1,001)
gross VAT credits	164,600	(243,600)	18,490	(23,410)	12,480	(20,980)	1,881	(14,610)	341	(1,013)
VAT due	34,040	(60,880)	7,677	(8,805)	3,734	(5,107)	605	(2,081)	121	(293)
new VAT credits	33,660	(103,000)	2,595	(11,400)	1,708	(6,588)	426	(12,010)	49	(488)
VAT payments	36,900	(72,290)	7,637	(9,355)	3,075	(5,355)	649	(11,230)	77	(218)
2018 e-invoices∗	0.85	(0.318)	0.91	(0.239)	0.94	(0.199)	0.93	(0.208)	0.32	(0.358)
VAT credit stock	21,070	(102,000)	1,818	(11,350)	2,044	(10,050)	592	(31,460)	68	(10,450)
exporting firms∗∗	0.63	(0.482)	0.49	(0.500)	0.37	(0.483)	0.12	(0.325)	0.07	(0.262)
number of workers	1,742	(2,816)	737	(1,309)	298	(487)	121	(634)	14	(43)
wage bill	11,160	(19,470)	2,322	(3,029)	906	(1,057)	344	(4,636)	25	(72)
capital	475,200	(896,100)	58,320	(186,700)	76,120	(561,000)	6,632	(77,820)	730	(7,425)
profits	118,100	(307,500)	14,690	(48,280)	9,511	(60,190)	1,276	(13,490)	183	(2,009)
										
observations	900		2,756		1,768		33,791		173,508	
percent of total V-A	37%		18%		6%		24%		16%	

**Note:** The balanced sample is constructed from the full sample by excluding wave 2 and wave 6 high-risk firms, firms not mandated by end 2017, as well as firms that have no sales in at least on quarter over 2013Q2–2017Q4. Waves correspond to groups of firms and are identified by initial deadline for e-invoicing adoption. Adoption deadlines are indicated in brackets at the column tops. Values correspond to annual averages and are expressed in thousands of 2014 soles, unless otherwise noted. In 2014, the exchange rate was approximately 0.34 US$ per soles. ∗ indicates that numbers correspond to the share of invoices issued electronically. ∗∗ indicates that numbers correspond to a dummy variables indicating if a firm exports.

**Table A.4:** Balanced sample summary statistics over 2014–2017: group distributions.

	Wave 1		Wave 3		Wave 4		Wave 5		Wave 7	
	obser-	value	obser-	value	obser-	value	obser-	value	obser-	value
(in % of total)	vation	added	vation	added	vation	added	vation	added	vation	added
Agriculture	1.3	0.8	0.6	0.5	0.5	0.3	3.2	4.7	1.9	2.9
Fishing	0.7	0.4	0.4	0.3	0.3	0.2	0.7	1.0	0.2	0.5
Extraction	18.6	28.6	7.8	9.3	5.7	27.5	1.6	9.5	0.5	1.5
Manufacturing	20.8	16.3	19.3	14.6	14.9	9.1	10.0	12.4	12.1	11.9
Utilities	4.0	4.1	1.5	1.6	0.7	0.2	0.9	3.1	0.2	0.5
Construction	3.4	2.4	7.5	8.5	12.0	8.7	5.4	4.1	4.8	9.1
Trade	33.0	8.9	22.8	10.1	27.3	15.7	35.3	11.7	38.9	22.3
Hotel-restaurant	0.2	0.1	1.7	0.9	2.2	1.3	3.6	1.5	4.0	3.3
transportation	3.0	1.5	4.1	6.6	6.1	4.5	13.4	5.7	10.5	7.4
Telecommunication	1.8	3.7	1.0	0.6	2.0	1.1	0.6	0.4	0.9	1.1
Other services	13.2	33.3	33.3	46.9	28.0	31.2	24.0	34.1	25.8	38.8
Public administration	0.0	0.0	0.0	0.0	0.2	0.1	1.2	11.6	0.1	0.5
										
SMEs										
- with no initial VAT credit stock	0.0	0.0	0.1	0.0	1.8	0.2	45.7	9.9	58.6	47.4
- with initial VAT credit stock	0.0	0.0	0.3	0.5	1.4	0.3	15.7	2.7	36.4	23.3
Large firms										
- with no initial VAT credit stock	53.8	48.8	72.1	73.1	55.9	67.2	27.2	45.4	2.6	13.8
- with initial VAT credit stock	46.2	51.2	27.4	26.4	41.0	32.3	11.4	41.9	2.4	15.5
										
Total (# obs; million soles)	900	344,038	2,756	163,580	1,768	53,956	33,791	222,795	173,508	146,746

**Note:** The balanced sample is constructed from the full sample by excluding wave 2 and wave 6 high-risk firms, firms not mandated by end 2017, as well as firms that have no sales in at least on quarter over 2013Q2–2017Q4. For every wave, the first column shows the percent of observations that belongs to the corresponding industry, size or VAT credit group. Waves correspond to groups of firms and are identified by initial deadline for e-invoicing adoption. Value added totals correspond to annual averages and are expressed in thousands of 2014 soles. In 2014, the exchange rate was approximately 0.34 US$ per soles. ∗ SMEs are firms with inflation-adjusted tax units between 150 and 2300, i.e. 0.6 and 8.7 million soles in 2014. ∗∗ refers to firms with a positive stock of VAT credits in 2013Q2.

**Table A.5:** Impact of mandatory e-Invoicing - robustness checks (1/4).

	(1)	(2)	(3)	(4)	(5)	(6)	(7)	(8)
	Taxable	Taxable	VAT	New VAT	VAT	Share of	Employed	Sales per
	sales	purchases	liabilities	credits	payments	taxable VA	workers	worker
**A. Baseline with firm-specific trends**								
Treatment	0.0743∗∗∗	0.0549∗∗∗	0.0820∗∗∗	0.0477	0.0544	−0.00216	0.0163∗∗∗	0.0439∗∗∗
(first year)	(0.0133)	(0.0146)	(0.0215)	(0.0390)	(0.0337)	(0.00522)	(0.00473)	(0.0106)
								
Dummy	0.158∗∗∗	0.123∗∗∗	0.309∗∗∗	−0.297∗∗	0.392∗∗∗	−0.00606	0.0266∗	0.106∗∗∗
(a year after)	(0.0277)	(0.0291)	(0.0793)	(0.140)	(0.0711)	(0.0216)	(0.0141)	(0.0232)
								
Constant	12.30∗∗∗	11.97∗∗∗	9.000∗∗∗	3.728∗∗∗	6.536∗∗∗	0.702∗∗∗	2.950∗∗∗	10.38∗∗∗
	(0.000753)	(0.000823)	(0.00126)	(0.00229)	(0.00192)	(0.000312)	(0.000290)	(0.000638)
								
Observations	1,010,380	1,010,380	1,010,380	1,010,380	1,010,380	1,010,380	936,527	936,527

**B. Alternative including the firms caught in fraudulent transactions before their assignment to a wave**								
Treatment	0.0706∗∗∗	0.0498∗∗∗	0.0860∗∗∗	0.0485	0.0530	−0.00233	0.0172∗∗∗	0.0436∗∗∗
(first year)	(0.0131)	(0.0144)	(0.0211)	(0.0382)	(0.0333)	(0.00508)	(0.00471)	(0.0106)
								
Dummy	0.160∗∗∗	0.127∗∗∗	0.252∗∗∗	−0.221∗	0.331∗∗∗	−0.00523	0.0157	0.121∗∗∗
(a year after)	(0.0271)	(0.0291)	(0.0708)	(0.126)	(0.0738)	(0.0189)	(0.0137)	(0.0234)
								
Constant	12.30∗∗∗	11.97∗∗∗	8.994∗∗∗	3.721∗∗∗	6.529∗∗∗	0.703∗∗∗	2.946∗∗∗	10.39∗∗∗
	(0.000784)	(0.000861)	(0.00132)	(0.00240)	(0.00201)	(0.000324)	(0.000301)	(0.000660)
								
Observations	1,015,622	1,015,622	1,015,622	1,015,622	1,015,622	1,015,622	939,663	939,663
**C. Alternative with wave trends**								
Treatment	0.0743∗∗∗	0.0548∗∗∗	0.0818∗∗∗	0.0476	0.0553∗	−0.00216	0.0104∗∗	0.0479∗∗∗
(first year)	(0.0129)	(0.0142)	(0.0209)	(0.0380)	(0.0328)	(0.00508)	(0.00462)	(0.0103)
								
Dummy	0.158∗∗∗	0.123∗∗∗	0.310∗∗∗	−0.297∗∗	0.393∗∗∗	−0.00606	0.0139	0.114∗∗∗
(a year after)	(0.0270)	(0.0284)	(0.0771)	(0.136)	(0.0692)	(0.0210)	(0.0138)	(0.0226)
								
Constant	12.30∗∗∗	11.97∗∗∗	9.000∗∗∗	3.728∗∗∗	6.536∗∗∗	0.702∗∗∗	2.950∗∗∗	10.38∗∗∗
	(0.000733)	(0.000801)	(0.00123)	(0.00223)	(0.00187)	(0.000304)	(0.000284)	(0.000622)
								
Observations	1,010,380	1,010,380	1,010,380	1,010,380	1,010,380	1,010,380	936,527	936,527

**Note:** These are results for the balanced sample of firms mandated to adopt e-invoicing before 2019, excluding firms previously caught for fraud except in Panel B. The last two columns drop firms with no reported workers. The inverse hyperbolic sine transformation is applied to all dependent variables, which are originally measured in constant 2014 Peruvian soles. The share of taxable VA is the ratio of taxable to total value added. The treatment indicator is equal to one in the quarter of mandatory e-invoicing adoption and the following four quarters. All specifications include a variable controlling for the fifth and following quarters after the date of mandatory e-invoicing adoption. They also include quarter fixed effects, firm fixed effects, firm-specific linear trends except in Panel C. Firm-clustered standard errors are shown in brackets. ∗ 0.10, ∗∗ 0.05, ∗∗∗ 0.01.

**Table A.6:** Impact of mandatory e-Invoicing - robustness checks (2/4).

	Taxable	Taxable	VAT	New VAT	VAT	Share of
	sales	purchases	liabilities	credits	payments	taxable VA
**D. Alternative on the subsample including non-missing employment observations**						
Treatment	0.0642∗∗∗	0.0447∗∗∗	0.0744∗∗∗	0.0496	0.0659∗	−0.00225
(first year)	(0.0131)	(0.0140)	(0.0215)	(0.0396)	(0.0340)	(0.00531)
						
Dummy	0.130∗∗∗	0.0844∗∗∗	0.275∗∗∗	−0.303∗∗	0.401∗∗∗	−0.00633
(a year after)	(0.0273)	(0.0279)	(0.0798)	(0.142)	(0.0703)	(0.0223)
						
Constant	12.44∗∗∗	12.10∗∗∗	9.154∗∗∗	3.710∗∗∗	6.789∗∗∗	0.705∗∗∗
	(0.000783)	(0.000839)	(0.00134)	(0.00246)	(0.00204)	(0.000336)
						
Observations	936,527	936,527	936,527	936,527	936,527	936,527
**E. Alternative with additional firm controls**						
Treatment	0.0569∗∗∗	0.0369∗∗∗	0.0679∗∗∗	0.0512	0.0556∗	−0.00221
(first year)	(0.0128)	(0.0137)	(0.0214)	(0.0396)	(0.0337)	(0.00531)
						
Dummy	0.116∗∗∗	0.0688∗∗	0.262∗∗∗	−0.303∗∗	0.381∗∗∗	−0.00622
(a year after)	(0.0263)	(0.0268)	(0.0797)	(0.142)	(0.0690)	(0.0223)
						
Number of	0.336∗∗∗	0.338∗∗∗	0.281∗∗∗	−0.151∗∗∗	0.437∗∗∗	0.00275
workers	(0.0115)	(0.0128)	(0.0199)	(0.0292)	(0.0272)	(0.00330)
						
Wage bill	0.0934∗∗∗	0.0939∗∗∗	0.137∗∗∗	−0.0242	0.170∗∗∗	−0.00571∗∗
	(0.00810)	(0.0103)	(0.0143)	(0.0208)	(0.0198)	(0.00232)
						
Capital stock	0.00585∗∗∗	0.00992∗∗∗	0.00217	0.0110∗∗∗	0.00882∗∗∗	−0.0000578
	(0.00122)	(0.00157)	(0.00196)	(0.00348)	(0.00333)	(0.000428)
						
Constant	10.42∗∗∗	10.02∗∗∗	6.890∗∗∗	4.274∗∗∗	3.637∗∗∗	0.756∗∗∗
	(0.0668)	(0.0852)	(0.116)	(0.171)	(0.164)	(0.0187)
						
Observations	936,527	936,527	936,527	936,527	936,527	936,527

**Note:** These are results for the balanced sample of firms mandated to adopt e-invoicing before 2019, excluding firms previously caught for fraud and excluding observations with zero or missing employment. The inverse hyperbolic sine transformation is applied to all dependent variables, which are originally measured in constant 2014 Peruvian soles. The share of taxable VA is the ratio of taxable to total value added. The treatment indicator is equal to one in the quarter of mandatory e-invoicing adoption and the following four quarters. All specifications include a variable controlling for the fifth and following quarters after the date of mandatory e-invoicing adoption and additionally controls for firm employment, wage bill and capital stock in Panel E. They also include quarter fixed effects, firm fixed effects, firm-specific linear trends. Firm-clustered standard errors are shown in brackets. ∗ 0.10, ∗∗ 0.05, ∗∗∗ 0.01.

**Table A.7:** Impact of mandatory e-Invoicing - robustness checks (3/4).

	(1)	(2)	(3)	(4)	(5)	(6)	(7)	(8)
	Taxable	Taxable	VAT	New VAT	VAT	Share of	Employed	Sales per
	sales	purchases	liabilities	credits	payments	taxable VA	workers	worker
**F. Alternative estimation sample with firms that enter and exit (unbalanced sample)**								
Treatment	0.318∗∗∗	0.169∗∗∗	0.318∗∗∗	0.00152	0.209∗∗∗	−0.00435	0.0331∗∗∗	0.144∗∗∗
(first year)	(0.0200)	(0.0165)	(0.0233)	(0.0357)	(0.0327)	(0.00473)	(0.00517)	(0.0147)
								
Dummy	0.692∗∗∗	0.408∗∗∗	0.789∗∗∗	−0.344∗∗	0.633∗∗∗	−0.0103	0.0558∗∗∗	0.286∗∗∗
(a year after)	(0.0606)	(0.0365)	(0.0858)	(0.134)	(0.0763)	(0.0204)	(0.0161)	(0.0371)
								
Constant	11.59∗∗∗	11.59∗∗∗	8.343∗∗∗	3.986∗∗∗	5.886∗∗∗	0.695∗∗∗	2.800∗∗∗	10.09∗∗∗
	(0.000890)	(0.000722)	(0.00105)	(0.00161)	(0.00143)	(0.000217)	(0.000256)	(0.000719)
								
Observations	1,495,662	1,495,662	1,495,662	1495662	1,495,662	1,495,662	1,285,523	1,285,523
								
**G. Alternative using annoucement dates instead of mandatory dates of adoption**								
Treatment	−0.0119	−0.0340∗∗∗	0.0140	−0.0734∗	−0.0492	0.00202	−0.0131∗∗∗	−0.00640
(first year)	(0.0127)	(0.0127)	(0.0235)	(0.0439)	(0.0326)	(0.00618)	(0.00417)	(0.0104)
								
Dummy	0.0611∗∗∗	0.0164	0.0818∗∗	−0.0846	0.0115	0.00258	0.00156	0.0353∗∗
(a year after)	(0.0202)	(0.0214)	(0.0363)	(0.0678)	(0.0528)	(0.00951)	(0.00713)	(0.0164)
								
Constant	12.30∗∗∗	11.97∗∗∗	9.001∗∗∗	3.736∗∗∗	6.543∗∗∗	0.702∗∗∗	2.952∗∗∗	10.38∗∗∗
	(0.00156)	(0.00161)	(0.00289)	(0.00540)	(0.00408)	(0.000763)	(0.000569)	(0.00136)
								
Observations	1,010,380	1,010,380	1,010,380	1,010,380	1,010,380	1,010,380	936,527	936,527

**Note:** These are results for the balanced sample of firms mandated to adopt e-invoicing before 2019, excluding firms previously caught for fraud. The last two columns drop firms with no reported workers. The inverse hyperbolic sine transformation is applied to all dependent variables, which are originally measured in constant 2014 Peruvian soles. The share of taxable VA is the ratio of taxable to total value added. The treatment indicator is equal to one in the quarter of mandatory e-invoicing adoption and the following four quarters. All specifications include a variable controlling for the fifth and following quarters after the date of mandatory e-invoicing adoption. They also include quarter fixed effects, firm fixed effects, firm-specific linear trends. Firm-clustered standard errors are shown in brackets. ∗ 0.10, ∗∗ 0.05, ∗∗∗ 0.01.

**Table A.8:** Impact of mandatory e-Invoicing - robustness checks (4/4).

	(1)	(2)	(3)	(4)	(5)	(6)	(7)	(8)
	Taxable	Taxable	VAT	New VAT	VAT	Share of	Employed	Sales per
	sales	purchases	liabilities	credits	payments	taxable VA	workers	worker
**H. Alternative excluding firms that adopted e-invoicing before adoption deadlines**								
Treatment	0.0803∗∗∗	0.0585∗∗∗	0.0818∗∗∗	0.0619	0.0528	−0.00383	0.0130∗∗	0.0501∗∗∗
(first year)	(0.0152)	(0.0168)	(0.0241)	(0.0435)	(0.0378)	(0.00585)	(0.00529)	(0.0122)
								
Dummy	0.168∗∗∗	0.122∗∗∗	0.338∗∗∗	−0.293∗	0.372∗∗∗	0.0104	0.0250∗	0.118∗∗∗
(a year after)	(0.0307)	(0.0314)	(0.0847)	(0.150)	(0.0766)	(0.0233)	(0.0151)	(0.0256)
								
Constant	12.17∗∗∗	11.89∗∗∗	8.846∗∗∗	3.754∗∗∗	6.270∗∗∗	0.700∗∗∗	2.880∗∗∗	10.35∗∗∗
	(0.000801)	(0.000884)	(0.00133)	(0.00239)	(0.00200)	(0.000330)	(0.000305)	(0.000684)
								
Observations	880,270	880,270	880,270	880,270	880,270	880,270	812,486	812,486
								
**I. Alternative excluding non-final consumer oriented sectors (80 percent threshold)**								
Treatment	0.0470∗	0.0254	0.136∗∗∗	0.121∗	0.119∗∗	−0.00303	0.00943	0.0327
(first year)	(0.0275)	(0.0312)	(0.0421)	(0.0719)	(0.0592)	(0.0110)	(0.00781)	(0.0210)
								
Dummy	0.123	0.198∗∗	0.544∗∗∗	0.0239	0.315∗∗	−0.0949	0.0325	0.118∗
(a year after)	(0.0858)	(0.0803)	(0.166)	(0.328)	(0.150)	(0.0591)	(0.0201)	(0.0679)
								
Constant	11.16∗∗∗	10.84∗∗∗	7.989∗∗∗	3.123∗∗∗	5.978∗∗∗	0.647∗∗∗	3.041∗∗∗	9.326∗∗∗
	(0.00146)	(0.00166)	(0.00231)	(0.00398)	(0.00314)	(0.000623)	(0.000454)	(0.00122)
								
Observations	243,337	243,337	243337	243,337	243,337	243,337	221,915	221,915
**J. Alternative excluding non-final consumer oriented sectors (60 percent threshold)**								
Treatment	0.0642∗∗∗	0.0318	0.144∗∗∗	0.0864	0.132∗∗	−0.00683	0.0194∗∗∗	0.0286∗
(first year)	(0.0213)	(0.0239)	(0.0341)	(0.0614)	(0.0545)	(0.00806)	(0.00692)	(0.0170)
								
Dummy	0.126∗∗	0.103	0.486∗∗∗	−0.459∗	0.379∗∗∗	−0.0800∗	0.0418∗∗	0.0923∗
(a year after)	(0.0609)	(0.0635)	(0.149)	(0.259)	(0.129)	(0.0437)	(0.0184)	(0.0504)
								
Constant	11.73∗∗∗	11.43∗∗∗	8.442∗∗∗	3.322∗∗∗	6.184∗∗∗	0.673∗∗∗	2.953∗∗∗	9.879∗∗∗
	(0.00112)	(0.00126)	(0.00183)	(0.00330)	(0.00287)	(0.000443)	(0.000391)	(0.000961)
								
Observations	391,176	391,176	391,176	391,176	391,176	391,176	358,335	358,335

**Note:** These are results for the balanced sample of firms mandated to adopt e-invoicing before 2019, excluding firms previously caught for fraud. The last two columns drop firms with no reported workers. The inverse hyperbolic sine transformation is applied to all dependent variables, which are originally measured in constant 2014 Peruvian soles. The treatment indicator is equal to one in the quarter of mandatory e-invoicing adoption and the following four quarters. All specifications include quarter fixed effects, firm fixed effects, firm-specific linear trends and a variable controlling for the fifth and following quarters after the date of mandatory e-invoicing adoption. Firm-clustered standard errors are shown in brackets. ∗ 0.10, ∗∗ 0.05, ∗∗∗ 0.01.

**Table A.9:** Heterogeneity by firm size.

	(1)	(2)	(3)	(4)	(5)	(6)	(7)
	Taxable	Taxable	VAT	New VAT	VAT	Employed	Sales per
	sales	purchases	liabilities	credits	payments	workers	worker
**Treatment (first year) interacted with**							
× SME dummy	0.106∗∗∗	0.101∗∗∗	0.116∗∗∗	0.0853∗	0.196∗∗∗	0.0246∗∗∗	0.0588∗∗∗
	(0.0183)	(0.0197)	(0.0251)	(0.0463)	(0.0465)	(0.00626)	(0.0149)
							
× large firm dummy	0.0379∗∗	0.00114	0.0432	0.00321	−0.109∗∗	0.00685	0.0268∗
	(0.0183)	(0.0204)	(0.0339)	(0.0608)	(0.0453)	(0.00670)	(0.0139)
**Treatment (after one year) interacted with**							
× SME dummy	−0.156	0.709∗∗	−0.430	1.762∗	0.599	0.179	0.440
	(0.524)	(0.314)	(0.853)	(0.952)	(0.655)	(0.137)	(0.349)
							
× large firm dummy	0.118∗∗∗	0.0533	0.270∗∗∗	−0.369∗∗	0.196∗∗	0.0144	0.0832∗∗∗
	(0.0311)	(0.0339)	(0.0850)	(0.151)	(0.0804)	(0.0154)	(0.0256)
							
Constant	12.30∗∗∗	11.97∗∗∗	9.000∗∗∗	3.728∗∗∗	6.538∗∗∗	2.950∗∗∗	10.38∗∗∗
	(0.000751)	(0.000826)	(0.00130)	(0.00236)	(0.00191)	(0.000293)	(0.000633)
							
Observations	1,010,380	1,010,380	1,010,380	1,010,380	1,010,380	936,527	936,527

**Note:** Results for the balanced sample of firms mandated to adopt e-invoicing before 2019. The inverse hyperbolic sine transformation is applied to all dependent variables, which are originally measured in constant 2014 Peruvian soles.The treatment indicator is equal to one in the quarter of mandatory e-invoicing adoption and the following four quarters. This treatment indicator is interacted with two dummy variables indicating if a firm had sales lower than 2,300 UIT (about $3 million) or a positive stock of VAT credits in 2013Q2, respectively. All specifications include quarter fixed effects, firm-specific trends and controls for the effects in the fifth and following quarters after the date of mandatory e-invoicing adoption. Firm-clustered standard errors are shown in brackets. ∗ 0.10, ∗∗ 0.05, ∗∗∗ 0.01. .

**Table A.10:** Heterogeneity by firm size and creditor status.

	(1)	(2)	(3)	(4)	(5)	(6)	(7)
	Taxable	Taxable	VAT	New VAT	VAT	Employed	Sales per
	sales	purchases	liabilities	credits	payments	workers	worker
**Treatment (first year) interacted with**							
× SME dummy ×	0.122∗∗∗	0.0940∗∗∗	0.166∗∗∗	−0.145∗∗∗	0.343∗∗∗	0.0198∗∗∗	0.0750∗∗∗
no VAT credit dummy	(0.0201)	(0.0217)	(0.0249)	(0.0494)	(0.0492)	(0.00710)	(0.0161)
							
× SME dummy ×	0.0611	0.123∗∗∗	−0.0278	0.747∗∗∗	−0.229∗∗	0.0389∗∗∗	0.0115
VAT credit dummy	(0.0393)	(0.0420)	(0.0629)	(0.101)	(0.105)	(0.0122)	(0.0326)
							
× large firm dummy ×	0.0626∗∗∗	0.0104	0.0844∗∗∗	−0.216∗∗∗	0.00277	0.0132∗	0.0359∗∗
no VAT credit dummy	(0.0214)	(0.0230)	(0.0302)	(0.0693)	(0.0504)	(0.00759)	(0.0161)
							
× large firm dummy ×	−0.0158	−0.0194	−0.0457	0.476∗∗∗	−0.353∗∗∗	−0.00681	0.00694
VAT credit dummy	(0.0343)	(0.0406)	(0.0839)	(0.116)	(0.0907)	(0.0131)	(0.0263)
**Treatment (after one year) interacted with**							
× SME dummy ×	−0.368	0.619	−0.241	0.391	−0.190	0.0181	−0.185
no VAT credit dummy	(0.582)	(0.535)	(0.597)	(0.952)	(0.934)	(0.122)	(0.217)
							
× SME dummy ×	0.0255	0.814∗∗	−0.721	3.612∗∗	1.096	0.323	0.930∗
VAT credit dummy	(0.882)	(0.321)	(1.602)	(1.506)	(0.810)	(0.213)	(0.532)
							
× large firm dummy ×	0.168∗∗∗	0.0495	0.414∗∗∗	−1.015∗∗∗	0.374∗∗∗	0.0442∗∗∗	0.0896∗∗∗
no VAT credit dummy	(0.0351)	(0.0410)	(0.0804)	(0.183)	(0.0852)	(0.0171)	(0.0289)
							
× large firm dummy ×	0.0134	0.0585	−0.0213	0.944∗∗∗	−0.176	−0.0460	0.0685
VAT credit dummy	(0.0601)	(0.0582)	(0.196)	(0.259)	(0.166)	(0.0305)	(0.0489)
							
Constant	12.30∗∗∗	11.97∗∗∗	9.000∗∗∗	3.728∗∗∗	6.538∗∗∗	2.950∗∗∗	10.38∗∗∗
	(0.000751)	(0.000827)	(0.00131)	(0.00235)	(0.00191)	(0.000293)	(0.000633)
							
Observations	1,010,380	1,010,380	1,010,380	1,010,380	1,010,380	936,527	936,527

**Note:** Results for the balanced sample of firms mandated to adopt e-invoicing before 2019. The inverse hyperbolic sine transformation is applied to all dependent variables, which are originally measured in constant 2014 Peruvian soles.The treatment indicator is equal to one in the quarter of mandatory e-invoicing adoption and the following four quarters. This treatment indicator is interacted with two dummy variables indicating if a firm had sales lower than 2,300 UIT (about $3 million) or a positive stock of VAT credits in 2013Q2, respectively. All specifications include quarter fixed effects, firm-specific trends and controls for the effects in the fifth and following quarters after the date of mandatory e-invoicing adoption. Firm-clustered standard errors are shown in brackets. ∗ 0.10, ∗∗ 0.05, ∗∗∗ 0.01. .

**Table A.11:** Heterogeneity by initial sales and creditor status (alternative specification).

	By Firm Size				By Firm Size and Creditor Status			
	Taxable	Taxable	New VAT	VAT	Taxable	Taxable	New VAT	VAT
	sales	purchases	credits	payments	sales	purchases	credits	payments
Treatment	0.106∗∗∗	0.109∗∗∗	0.101∗∗∗	0.214∗∗∗	0.0992∗∗∗	0.108∗∗∗	0.0823∗∗∗	0.169∗∗∗
(first year)	(0.0189)	(0.0217)	(0.0274)	(0.0475)	(0.0194)	(0.0223)	(0.0288)	(0.0493)
**Interacted with:**								
× Initial sales	−0.0293∗∗∗	−0.0501∗∗∗	−0.0185	−0.148∗∗∗	−0.0244∗∗	−0.0499∗∗∗	−0.00550	−0.118∗∗∗
	(0.0110)	(0.0139)	(0.0215)	(0.0253)	(0.0113)	(0.0141)	(0.0205)	(0.0261)
								
× VAT credit					−0.0282∗∗	−0.00129	−0.0761∗∗∗	−0.176∗∗∗
stock indicator					(0.0129)	(0.0134)	(0.0265)	(0.0313)
								
Dummy	0.0723	0.380∗∗∗	0.556	0.791∗∗∗	0.0578	0.386∗∗∗	0.507	0.752∗∗∗
(a year after)	(0.151)	(0.0909)	(0.346)	(0.240)	(0.151)	(0.0907)	(0.345)	(0.243)
**Interacted with:**								
× Initial sales	0.00826	−0.129∗∗∗	−0.102	−0.254∗∗∗	0.0195	−0.133∗∗∗	−0.0643	−0.220∗∗∗
	(0.0516)	(0.0336)	(0.126)	(0.0817)	(0.0528)	(0.0339)	(0.126)	(0.0833)
								
× VAT credit					−0.0657∗∗∗	0.0180	−0.213∗∗∗	−0.231∗∗∗
stock indicator					(0.0252)	(0.0215)	(0.0720)	(0.0576)
								
Constant	12.30∗∗∗	11.97∗∗∗	9.001∗∗∗	6.539∗∗∗	12.30∗∗∗	11.97∗∗∗	9.001∗∗∗	6.539∗∗∗
	(0.000747)	(0.000832)	(0.00139)	(0.00188)	(0.000748)	(0.000832)	(0.00139)	(0.00188)
								
Observations	1,010,333	1,010,333	1,010,333	1,010,333	1,010,333	1,010,333	1,010,333	1,010,333

**Note:** Regression results for the balanced sample of firms mandated to adopt e-invoicing before 2019. The inverse hyperbolic sine transformation is applied to all dependent variables, which are originally measured in constant 2014 Peruvian soles. Treatment, the main regressor, is equal to one in the quarter of mandatory e-invoicing adoption and the following four quarters. The treatment variable is interacted with log sales in 2013Q2 and with the log of one plus the stock of VAT credits in 2013Q2. Both interaction terms were demeaned and normalized to facilitate interpretation. All specifications include quarter fixed effects, firm-specific trends and a variable controlling for the fifth and following quarters after the date of mandatory e-invoicing adoption as well as corresponding interaction terms. Firm-clustered standard errors in brackets. ∗ 0.10, ∗∗ 0.05, ∗∗∗ 0.01.

**Table A.12:** Instrumental variable results.

	1st Stage	2nd Stage				
	Adoption	Taxable	Taxable	VAT	New VAT	VAT
		sales	purchases	liabilities	credits	payments
Treatment	0.448∗∗∗					
(first year)	(0.00396)					
						
Adoption		0.166∗∗∗	0.123∗∗∗	0.183∗∗∗	0.106	0.121
		(0.0297)	(0.0325)	(0.0480)	(0.0872)	(0.0753)
						
Dummy	0.463∗∗∗	0.0811∗∗∗	0.0659∗∗∗	0.224∗∗∗	−0.346∗∗∗	0.336∗∗∗
(a year after)	(0.00735)	(0.0219)	(0.0223)	(0.0748)	(0.132)	(0.0556)
Robust F-stat	1.30E+04					
Observations	1,010,380	1,010,380	1,010,380	1,010,380	1,010,380	1,010,380

**Note:** Regression results for the balanced sample of firms mandated to adopt e-invoicing before 2019. The inverse hyperbolic sine transformation is applied to all dependent variables, which are originally measured in constant 2014 Peruvian soles. The treatment indicator is equal to one in the quarter of mandatory e-invoicing adoption and the following four quarters. All specifications control include quarter fixed effects, firm-specific trends and a variable controlling for the fifth and following quarters after the date of mandatory e-invoicing adoption. Firm-clustered standard errors are shown in brackets. ∗ 0.10, ∗∗ 0.05, ∗∗∗ 0.01.

## Appendix A Additional tables and figures

See Fig. A.1, Fig. A.2, Fig. A.3, Fig. A.4, Fig. A.5, Fig. A.6, Fig. A.7 and Table A.1, Table A.2, Table A.3, Table A.4, Table A.5, Table A.6, Table A.7, Table A.8, Table A.9, Table A.10, Table A.11, Table A.12.

## Footnotes

1 In addition to their negative impact on tax collection, non-refundable VAT credits can also lower firm investment. See, for example, Chen et al. (2020) in the case of China.

2 Unintentional mistakes in tax filing and lack of information are an important driver of compliance, especially among smaller firms. For example, Kosonen and Ropponen (2015) find that highlighting a recent VAT change in Finland in the context of a taxpayer survey, lowered noncompliance among firms by more than 50 percent.

3 Even in these cases, firms were required to regularize paper invoices on a timely basis (RS 113-2018/SUNAT).

4 Adoption by large firms can have positive network spillovers, if it encourages clients and suppliers to transition to e-invoicing as well. It can introduce a bias when comparing mandated to not-yet mandated firms if a non-negligible share of not-yet mandated firms voluntarily adopt e-invoicing in advance. However, the bias works against identifying any effects and our results should therefore be interpreted as a lower bound of the real effect of e-invoicing. Furthermore, we show that voluntary adoption before the slated deadlines was rare.

5 See RS 374-2013/SUNAT for the original deadline, and RS 300-2014/SUNAT and RS 086-2015/SUNAT for deadline extensions.

6 See RS 300-2014/SUNAT.

7 The first group was drawn from the large firms register as of 30 September 2014 (Intendencia de Principales Contribuyentes Nacionales - IPCN) and was given an original deadline of 1st July 2015 (RS 300-2014/SUNAT), which was later extended until 1st January 2016 (RS 137-2015/SUNAT). The second group included new additions to the IPCN registry as of 31 July 2015. Its original deadline was 15th July 2016 (RS 203-2015/SUNAT), but this was later extended by six months (RS 328-2016/SUNAT).

8 Three special VAT withholding regimes are currently used in the Peru, aimed at improving tax compliance in certain industries or transactions. The “Retention” mechanism requires some taxpayers to collect part of the VAT liabilities of their suppliers, while the “Perception” mechanism works in reverse by forcing certain suppliers to pay additional tax as part of their customers’ VAT liabilities. In both cases, suppliers or customers can later deduct the withheld amount from their tax liabilities. A third “Deposit” mechanism (SPOT) requires purchasers of specific goods and services to deposit a percentage of that transaction value into a bank account under the name of their supplier. Suppliers use these accounts to offset future tax liabilities.

9 See RS 203-2015/SUNAT for the original deadline, and RS 311-2016/SUNAT and RS 155-2017/SUNAT for the subsequent extensions. This group was drawn from registries of small and medium-sized firms (Principales Contribuyentes - PRICOS) for Lima and other provinces in Peru, as of 31st July 2017.

10 See Fig. A.2 in appendix for more details.

11 See footNote 8 for a brief description of these withholding regimes.

12 See RS 192-2016/SUNAT. The latter group included all firms registered in the IPCN registry as of 29 June 2017, but that had not been included in previous waves (RS 155-2017/SUNAT).

13 See Fig. A.2 in appendix for more details about the deadline extensions for specific waves.

14 It is also likely that some firms made the switch voluntarily to take advantage of lower processing costs, or to facilitate transactions with their suppliers or clients that had already adopted e-invoices.

15 The departure of VAT from a perfectly enforced tax levied at a uniform rate on all consumption can be measured by its “C-efficiency”, the ratio of actual VAT revenue to the product of the standard rate and consumption as taken from national accounts. Keen (2013) decomposes this indicator further into a policy gap, reflecting rate differentiation and exemptions, and a compliance gap, which measures imperfect implementation of the VAT. Since 2005, the compliance gap in Peru has ranged between 30 and 40 percent of potential VAT revenues, higher than the average across Latin American countries (IMF, 2015).

16 The Unidad Impositiva Tributaria (UIT) is a monetary unit set every year by the tax authority to calculate tax and regulatory thresholds. For example, firms with less than 150 UIT in annual sales are defined as micro firms in the Peruvian legal system. In addition, firms with less than 96,000 soles (about $29,000) in annual sales were automatically excluded from our analysis because they are subject to a simplified tax regime in Peru (the Nuevo RUS), instead of the VAT regime.

17 The dataset was constructed by including all the firms that reported annual sales above 150 UIT at least once over the 2010–2017 period.

18 INEI (2020) estimates that informal firms represent about 20 percent of total value added.

19 All the variables considered are non-negative but the transformation ensures that we keep zeros. We examine an alternative log transformation (y→ln(y+1)) and found very similar results. These results are available upon request.

20 Since the treatment variable takes only the values zero or one, the percentage change in the variable of interest can be calculated as $\frac{\text{arcsinh}\;Y^{T=1}-\text{arcsinh}\;Y^{T=0}}{\text{arcsinh}\;Y^{T=0}}\approx \exp\beta -1$, where the approximation holds when *Y* is large. This is also the standard result for logarithmic equations (Bellemare and Wichman, 2020).

21 The estimation sample becomes smaller as we drop observations with missing employment values. We verify in Table A.6 Panel D in the appendix that employment results are not driven by composition effects as the first six coefficient in Table 1 are estimated to remain essentially the same on the subsample with non-zero employment observation.

22 This last category includes financial, consulting, IT, marketing, accounting and legal, rental, architecture, research, and engineering services. We exclude agriculture, fishing, utilities, telecommunication and public administration from the analysis because these sectors accounted for less than 5 percent of the observations or the total value added in our sample. We also exclude mining since it is dominated by a few large firms that are primarily exporters.
