# Supplementary material for: Digitalization to improve tax compliance: Evidence from VAT e-Invoicing in Peru
Source: J Public Econ. 2022 Jun;210:104661. doi: 10.1016/j.jpubeco.2022.104661 (PMC9168443; doi:10.1016/j.jpubeco.2022.104661)
Supplement: Supplementary data 1 [file mmc1.pdf]

Supplementary Appendix for
Digitalization to Improve Tax Compliance: Evidence from VAT e-Invoicing in Peru

Matthieu Bellon, Era Dabla-Norris, Salma Khalid and Frederico Lima*

July 15, 2021

B Supplementary Tables and Figures

*Bellon: mbellon@imf.org; Dabla-Norris: edablanorris@imf.org; Khalid: skhalid@imf.org; Lima: flima@imf.org; The views expressed herein are those of the authors and should not be attributed to the IMF, its Executive Board, or its management.

TABLE B.1: Impact of Mandatory e-Invoicing - Robustness Checks 5

	(1)	(2)	(3)	(4)	(5)	(6)	(7)	(8)
	Taxable sales	Taxable purchases	VAT liabilities	New VAT credits	VAT payments	Taxable VA	Employed workers	Sales per worker
**K. Alternative sample excluding wave 3**								
Treatment	0.0843***	0.0631***	0.0967***	0.0369	0.0658*	0.124*	0.0188***	0.0497***
(first year)	(0.0141)	(0.0154)	(0.0217)	(0.0397)	(0.0357)	(0.0711)	(0.00498)	(0.0112)
Dummy	0.167***	0.109**	0.231*	-0.142	0.228**	0.953**	0.00713	0.137***
(a year after)	(0.0352)	(0.0444)	(0.128)	(0.206)	(0.0948)	(0.427)	(0.0194)	(0.0302)
Constant	12.23***	11.90***	8.943***	3.702***	6.439***	7.149***	2.904***	10.36***
	(0.000684)	(0.000749)	(0.00108)	(0.00196)	(0.00174)	(0.00352)	(0.000259)	(0.000578)
Observations	997,289	997,289	997,289	997,289	997,289	997,289	923,757	923,757
**L. Alternative using the log transformation instead of the inverse hyerbolic sine one**								
Treatment	0.0843***	0.0631***	0.0967***	0.0369	0.0658*	0.0653***	0.0188***	0.0497***
(first year)	(0.0141)	(0.0154)	(0.0217)	(0.0397)	(0.0357)	(0.0123)	(0.00498)	(0.0112)
Dummy	0.167***	0.109**	0.231*	-0.142	0.228**	0.170***	0.00713	0.137***
(a year after)	(0.0352)	(0.0444)	(0.128)	(0.206)	(0.0948)	(0.0266)	(0.0194)	(0.0302)
Constant	12.23***	11.90***	8.943***	3.702***	6.439***	10.48***	2.904***	10.36***
	(0.000684)	(0.000749)	(0.00108)	(0.00196)	(0.00174)	(0.000703)	(0.000259)	(0.000578)
Observations	997,289	997,289	997,289	997,289	997,289	843,101	923,757	923,757
**M. Alternative excluding voluntary adopters from the control group**								
Treatment	0.0756***	0.0574***	0.0797***	0.0470	0.0451	0.0801	0.0132***	0.0479***
(first year)	(0.0134)	(0.0146)	(0.0217)	(0.0394)	(0.0339)	(0.0705)	(0.00475)	(0.0107)
Dummy	0.161***	0.128***	0.305***	-0.298**	0.372***	0.708***	0.0198	0.114***
(a year after)	(0.0279)	(0.0293)	(0.0794)	(0.140)	(0.0716)	(0.262)	(0.0141)	(0.0233)
Constant	12.25***	11.95***	8.926***	3.748***	6.418***	7.065***	2.945***	10.36***
	(0.000833)	(0.000911)	(0.00140)	(0.00254)	(0.00212)	(0.00457)	(0.000321)	(0.000708)
Observations	918,745	918,745	918,745	918,745	918,745	918,745	850,279	850,279

Note: These are results for the balanced sample of firms mandated to adopt e-invoicing before 2019, excluding firms previously caught for fraud. The last two columns drop firms with no reported workers. All dependent variables y are originally measured in constant 2014 soles (local currency) and transformed using $\ln(1+x)$ in panels L and M, and using $\ln(x+\sqrt{x^2+1})$ in panel K. The treatment indicator is equal to one in the quarter of mandatory e-invoicing adoption and the following four quarters. All specifications include a variable controlling for the fifth and following quarters after the date of mandatory e-invoicing adoption. They also include quarter fixed effects, firm fixed effects, and firm-specific trends. Firm-clustered standard errors are shown in brackets. * 0.10, ** 0.05, *** 0.01.

Table B.2: Impact of Mandatory e-Invoicing by Wave of Adoption Requirements (1/2)

	(1)	(2)	(3)	(4)	(5)	(6)	(7)	(8)
	Taxable sales	Taxable purchases	VAT liabilities	New VAT credits	VAT payments	Share of taxable VA	Employed workers	Sales per worker
**Wave 1**								
Treatment	-0.00649	-0.0713	0.423*	-0.257	0.148	-0.00699	0.0239*	-0.0397
(first year)	(0.0362)	(0.0574)	(0.219)	(0.462)	(0.130)	(0.0744)	(0.0129)	(0.0367)
Constant	11.98***	11.71***	8.665***	3.753***	5.769***	0.712***	2.650***	10.39***
	(0.0000848)	(0.000135)	(0.000513)	(0.00108)	(0.000306)	(0.000175)	(0.0000328)	(0.0000930)
Observations	479,594	479,594	479,594	479,594	479,594	479,594	431,629	431,629
**Wave 2**								
Treatment	-0.0190	-0.0713	0.0545	-0.0530	0.294	-0.00383	-0.0451	0.0726
(first year)	(0.0701)	(0.0828)	(0.129)	(0.272)	(0.275)	(0.0129)	(0.0421)	(0.109)
Constant	11.97***	11.70***	8.682***	3.709***	5.805***	0.715***	2.629***	10.39***
	(0.000163)	(0.000192)	(0.000300)	(0.000631)	(0.000638)	(0.0000301)	(0.0000717)	(0.000186)
Observations	479,571	479,571	479,571	479,571	479,571	479,571	433,747	433,747
**Wave 3**								
Treatment	-0.0519***	-0.0690***	-0.0415	0.324	-0.0298	-0.00724	-0.0102	-0.0370**
(first year)	(0.0177)	(0.0268)	(0.116)	(0.237)	(0.0579)	(0.0355)	(0.00850)	(0.0174)
Constant	12.10***	11.81***	8.820***	3.685***	6.104***	0.712***	2.709***	10.42***
	(0.000126)	(0.000190)	(0.000825)	(0.00169)	(0.000411)	(0.000252)	(0.0000642)	(0.000131)
Observations	484,702	484,702	484,702	484,702	484,702	484,702	444,481	444,481

Note: These are results by wave adoption requirements for the balanced sample of firms mandated to adopt e-invoicing before 2019. Each panel is run on the 11-quarter window around the mandatory date of adoption and include the wave of interest as well as the control group of firms mandated after 2017. The last two columns drop firms with no reported workers. The inverse hyperbolic sine transformation is applied to all dependent variables, which are originally measured in constant 2014 soles (local currency). The share of taxable VA is the ratio of taxable to total value added. The treatment indicator is equal to one in the quarter of mandatory e-invoicing adoption and the following four quarters. All specifications include quarter fixed effects, firm fixed effects and firm-specific trends. Firm-clustered standard errors are shown in brackets. * 0.10, ** 0.05, *** 0.01.

Table B.3: Impact of Mandatory e-Invoicing by Wave of Adoption Requirements (2/2)

	(1)	(2)	(3)	(4)	(5)	(6)	(7)	(8)
	Taxable sales	Taxable purchases	VAT liabilities	New VAT credits	VAT payments	Share of taxable VA	Employed workers	Sales per worker
**Wave 4**								
Treatment	0.00740	0.0195	-0.134	0.319	-0.116	-0.00677	-0.00877	0.0228
(first year)	(0.0634)	(0.0439)	(0.171)	(0.291)	(0.118)	(0.0411)	(0.0190)	(0.0483)
Constant	12.11***	11.80***	8.846***	3.660***	6.250***	0.712***	2.696***	10.42***
	(0.000291)	(0.000201)	(0.000782)	(0.00133)	(0.000542)	(0.000189)	(0.0000891)	(0.000226)
Observations	481,985	481,985	481,985	481,985	481,985	481,985	447,895	447,895
**Wave 5**								
Treatment	0.0494***	0.0450***	0.0816***	0.0371	-0.0413	0.000644	0.0143***	0.0318**
(first year)	(0.0157)	(0.0154)	(0.0275)	(0.0554)	(0.0409)	(0.00762)	(0.00395)	(0.0127)
Constant	12.23***	11.87***	8.977***	3.610***	6.607***	0.705***	2.878***	10.36***
	(0.00116)	(0.00114)	(0.00204)	(0.00410)	(0.00303)	(0.000565)	(0.000306)	(0.000980)
Observations	570,048	570,048	570,048	570,048	570,048	570,048	535,566	535,566
**Wave 6**								
Treatment	0.232	0.186	0.127	0.426	0.134	-0.0152	0.0437	0.369
(first year)	(0.279)	(0.202)	(0.314)	(0.493)	(0.625)	(0.0371)	(0.108)	(0.421)
Constant	12.09***	11.77***	8.835***	3.644***	6.251***	0.713***	2.670***	10.42***
	(0.000136)	(0.0000988)	(0.000153)	(0.000241)	(0.000305)	(0.0000181)	(0.0000330)	(0.000129)
Observations	434,276	434,276	434,276	434,276	434,276	434,276	404,495	404,495

Note: These are results by wave adoption requirements for the balanced sample of firms mandated to adopt e-invoicing before 2019. Each panel is run on the 11-quarter window around the mandatory date of adoption and include the wave of interest as well as the control group of firms mandated after 2017. The last two columns drop firms with no reported workers. The inverse hyperbolic sine transformation is applied to all dependent variables, which are originally measured in constant 2014 soles (local currency). The share of taxable VA is the ratio of taxable to total value added. The treatment indicator is equal to one in the quarter of mandatory e-invoicing adoption and the following four quarters. All specifications include quarter fixed effects, firm fixed effects and firm-specific trends. Firm-clustered standard errors are shown in brackets. * 0.10, ** 0.05, *** 0.01.

Figure B.1: Impact of e-Invoicing Around the Mandatory Date of Adoption - Alternative Specification with Wave Trends

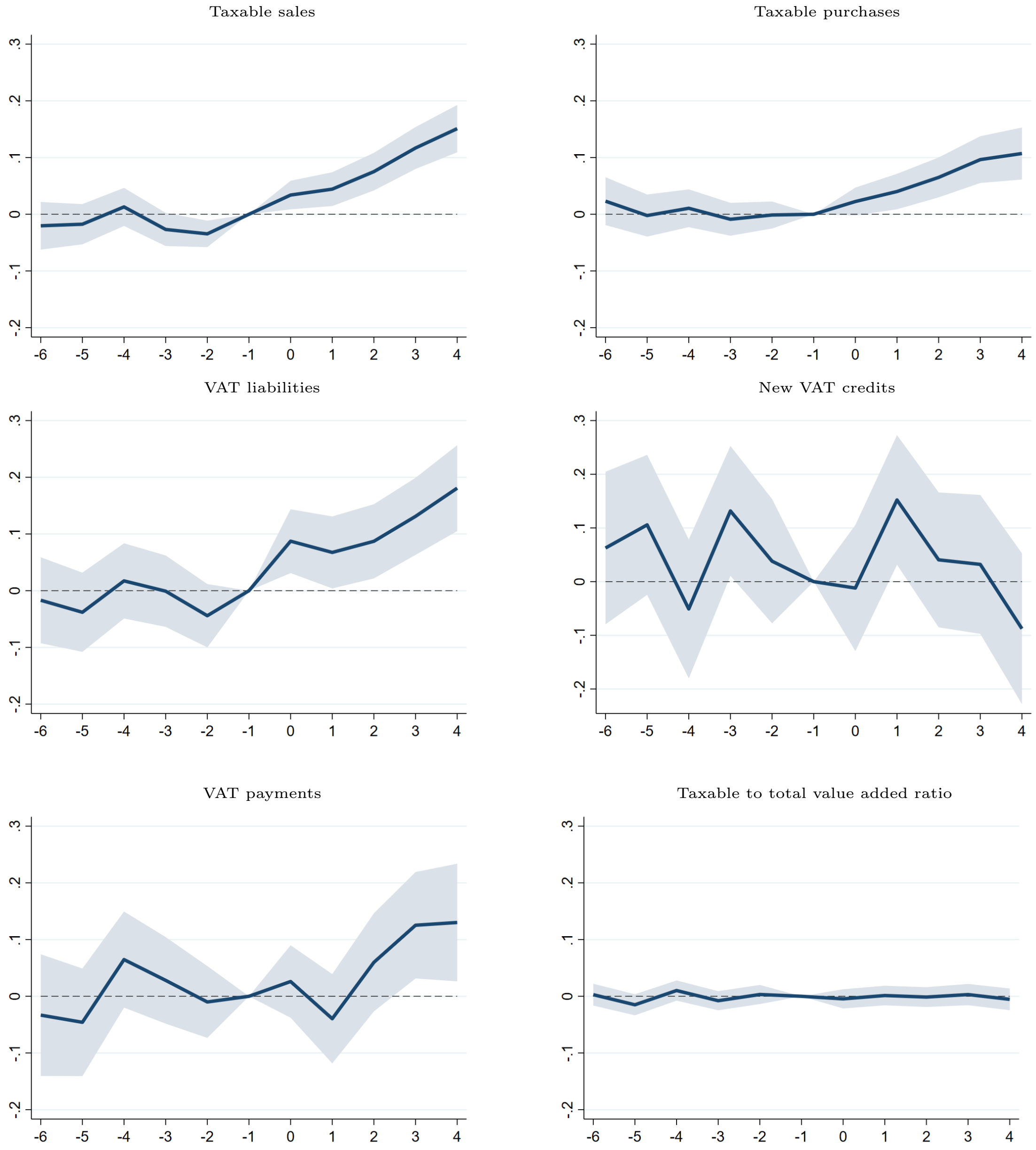

Note: This figure plots the point estimates (solid line) and the 95 percent confidence intervals (shaded area) of the β_t coefficients in Equation 2, except that wave trends are used instead of firm-specific trends. The results are obtained using the balanced sample of firms mandated to adopt e-invoicing before 2019. The responses represent the percent change in the dependent variable relative to the mandatory adoption quarter.

FIGURE B.2: Impact of e-Invoicing Around the Mandatory Date of Adoption - Alternative Specification with No Trends

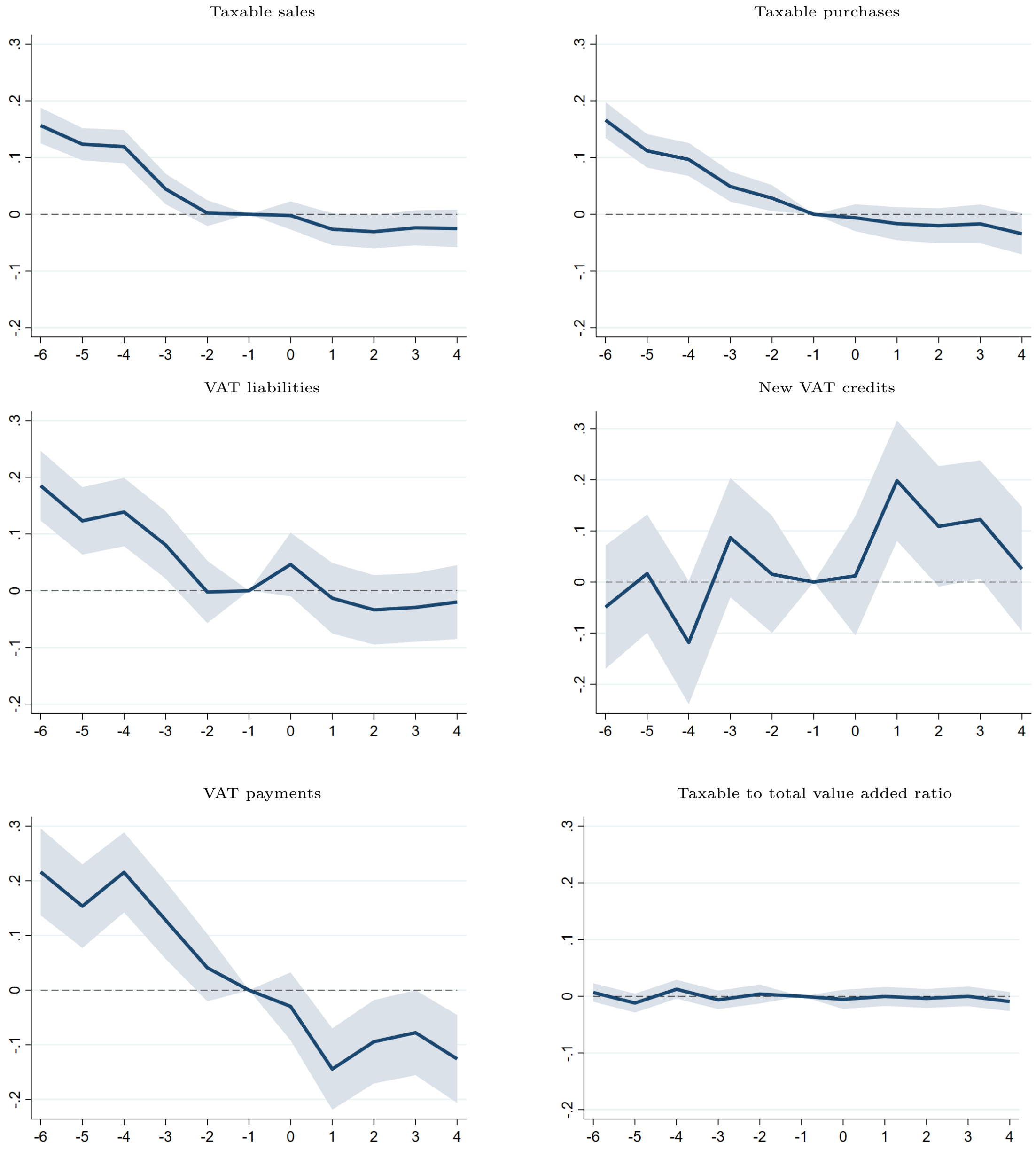

Note: This figure plots the point estimates (solid line) and the 95 percent confidence intervals (shaded area) of the β_t coefficients in Equation 2, except that we dropped firm-specific trends. The results are obtained using the balanced sample of firms mandated to adopt e-invoicing before 2019. The responses represent the percent change in the dependent variable relative to the mandatory adoption quarter.

FIGURE B.3: Impact of e-Invoicing Around the Mandatory Date of Adoption - Alternative Specification with ONR Waves

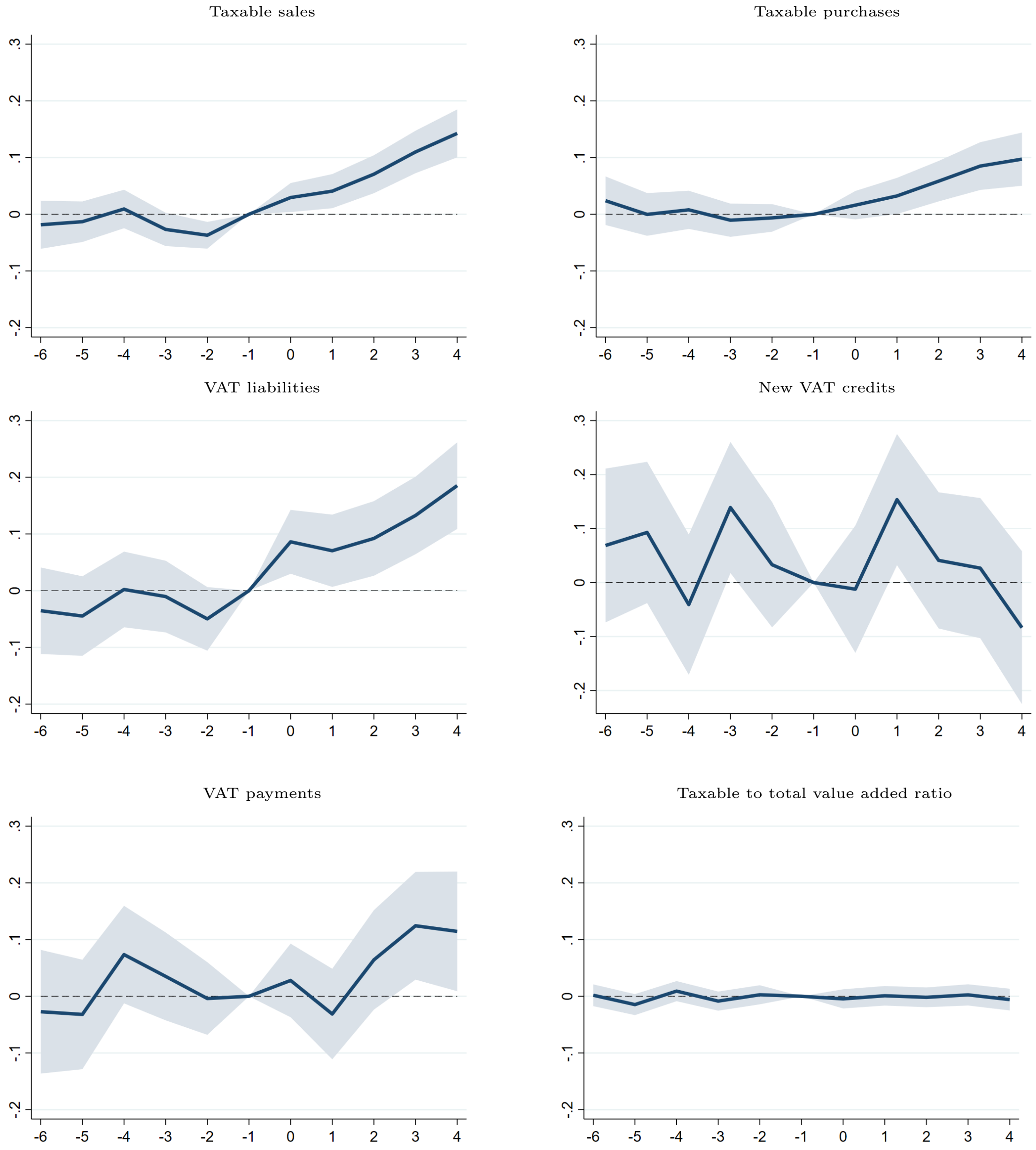

Note: This figure plots the point estimates (solid line) and the 95 percent confidence intervals (shaded area) of the β_t coefficients in Equation 2. The estimation sanmple includes firms from the "ONR waves" 2 and 4 with firms that were caugfht in fraudulent transactions before being assigned an adoption deadline. The results are obtained using the balanced sample of firms mandated to adopt e-invoicing before 2019. The responses represent the percent change in the dependent variable relative to the mandatory adoption quarter.

Figure B.4: Impact of e-Invoicing Around the Mandatory Date of Adoption by wave

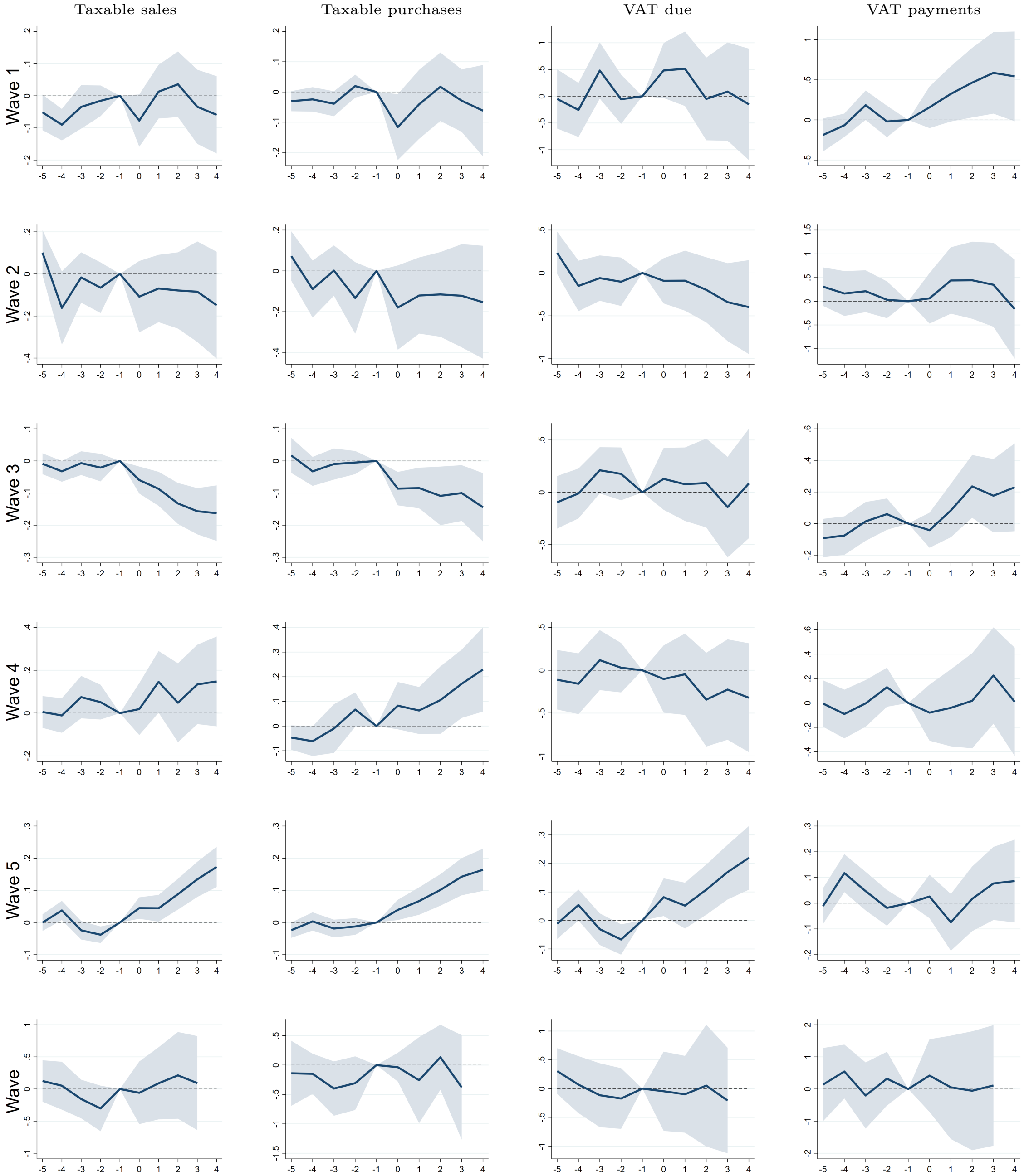

Note: Each row corresponds to a wave of firms with a specific adoption deadline. The estimation sample is restricted to the firms in the wave of interest and those in the control group (that is firms mandated in 2018) and only includes an 11-quarter window centered around the adoption deadlines. The responses represent the percent change in the dependent variable relative to the mandatory adoption quarter.

Figure B.5: E-Invoicing Impact Around Announcement Dates and Adoption Deadlines - Excluding Firms in Wave 3

A) Around the mandatory dates of adoption
Survival indicator

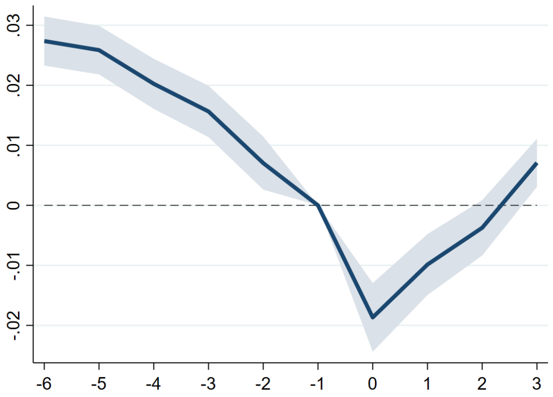

B) Around the announcement dates of deadlines
Survival indicator

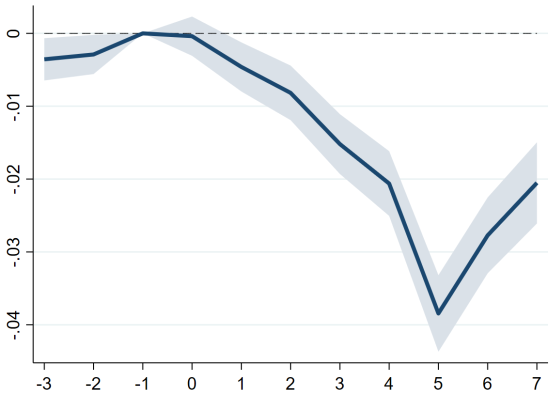

Note: In the (A) panels, the reference period -1 corresponds to the quarter before the deadline for adopting e-invoicing. In (B) panels, instead of using the deadlines for adoption as before, the graph shows the relatives changes of the treated around the *announcement dates*, that is when deadlines were announced. The dependent variable is a firm indicator of survival that takes the value one until the last quarter where positive sales are observed and the value zero from then on to the end of the sample; the estimation sample excludes the last quarter (2017Q4) because survival cannot be computed then. The estimation sample excludes firm in wave 3. This figure plots the point estimates (solid line) and the 95 percent confidence intervals (shaded area) of the β_t coefficients in Equation 2.
